# Changing lanes: extending CAR T-cell therapy to high-risk plasma cell dyscrasias

**DOI:** 10.3389/fimmu.2025.1558275

**Published:** 2025-04-08

**Authors:** Heather T. Morgan, Benjamin A. Derman, Hong Ma, Shaji K. Kumar

**Affiliations:** ^1^ Clinical Development, Oricell Therapeutics, Roseland, NJ, United States; ^2^ Section of Hematology/Oncology, University of Chicago, Chicago, IL, United States; ^3^ Department of Hematology, Mayo Clinic, Rochester, MN, United States

**Keywords:** systemic light chain amyloidosis, AL amyloidosis, plasma cell leukemia, CAR T-cell, plasma cell dyscrasia, multiple myeloma, immunotherapy

## Abstract

Chimeric antigen receptor (CAR) cellular therapies have advanced outcomes in challenging hematologic malignancies like leukemia, lymphoma, and multiple myeloma. Plasma cell-directed CAR T-cell therapies have been particularly beneficial in multiple myeloma, suggesting that these agents may have a role in other challenging plasma cell disorders such as systemic AL amyloidosis and plasma cell leukemia. AL amyloidosis is a monoclonal plasma cell disorder resulting in the deposition of protein fibrils that compromise end-organ function. Delays in diagnosis can result in end-organ dysfunction and organ failure, making designing and completing treatment difficult. Plasma cell leukemia (PCL) is a rare and highly challenging malignancy with dismal survival outcomes despite aggressive therapy. Both diagnoses are currently treated with regimens borrowed from myeloma: a combination of novel agents and chemotherapy induction, then autologous stem cell transplantation (ASCT), with the current practice trending towards consolidation and maintenance. Unfortunately, only 20% of AL amyloidosis patients are transplant-eligible at diagnosis. Those transplant-ineligible (TIE) patients are treated with combination induction chemotherapy, which may be limited by worsening disease-related end-organ dysfunction. Plasma cell leukemia patients are still very likely to relapse after this intensive and prolonged therapy. Despite the promise of a shorter course of therapy, CAR T-cell therapies directed against plasma cells have not been rigorously investigated in patients with AL amyloidosis or PCL; most trials of MM have excluded these patients. Herein, we describe current treatment paradigms for AL amyloidosis and PCL and review the evidence for CAR T-cell therapies in these challenging plasma cell disorders. Further investigation into CAR T-cell therapies for plasma cell disorders other than multiple myeloma is warranted.

## 1 Introduction

The development of chimeric antigen receptor (CAR) T-cell therapies has revolutionized the treatment approach for many recalcitrant malignancies (1–3). The evolution and expansion of cell therapies from leukemia into lymphoma and multiple myeloma (MM) has yielded knowledge and experience that provide a foundation for subsequent areas of clinical development (4–8). CAR T-cell therapies are expanding across indications, including other plasma cell dyscrasias and solid tumors (9–15). These investigations offer invaluable instruction on cancer development and tumor microenvironment, prompting further study. What began less than 10 years prior as a high-risk therapy characterized by dangerous and unmanageable side effects that were poorly understood has become a standardized treatment modality used worldwide (16–21).

In MM, there are two CAR T-cell therapy products with U.S. Food and Drug Administration (FDA) approval: idecabtagene vicleucel (ide-cel, Abecma) and ciltacabtagene autoleucel (cilta-cel, Carvykti). Both are directed against B-cell maturation antigen (BCMA) and gained FDA approval in March 2021 and February 2022, respectively (22, 23). Idecabtagene vicleucel (ide-cel) was initially approved based on the results of the KarMMa study for the treatment of patients with relapsed or refractory MM after four lines of therapy with prior exposure to an immunomodulatory drug (IMiD), proteasome inhibitor (PI), and anti-CD38 monoclonal antibody (mAb). This indication was advanced in March 2024 to include patients with two lines of therapy after the phase 3 KarMMa-3 study showed significantly prolonged progression-free survival (PFS) and improved response compared to standard treatment, with a comparable safety profile to previous results (24). Ciltacabtagene autoleucel (cilta-cel) was approved for relapsed or refractory MM after four lines of therapy with prior exposure to an IMiD, PI, and anti-CD38 monoclonal antibody, based on the results of the CARTITUDE-1 study (25). The phase 3 CARTITUDE-3 study showed significantly improved overall response rates, PFS, minimal residual disease (MRD) negativity, and lower risk of death than the standard of care (26). Based on these results, the indication for cilta-cel was also expanded in 2024 to include patients with lenalidomide-refractory MM who had received at least one prior line of therapy that included a PI.

The successes seen in MM have prompted further study of CAR T-cells in patients with newly diagnosed MM (24, 26–30). This raises the question of whether these existing plasma-cell-directed CAR T therapies could benefit other challenging plasma cell disorders, particularly those without a clear standard of care or with inferior outcomes (31–35).

Historically, most rare plasma cell disorders like light chain amyloidosis (AL amyloidosis) and plasma cell leukemia (PCL) have been excluded from clinical trials in MM, which has limited progress in these diseases. Some advances have been made with the adoption of intensive MM-like regimens. Still, outcomes remain poor, particularly for those with advanced disease, those transplant-ineligible, the elderly, and those with significant end-organ dysfunction (36–38). Here, we review the currently available data for AL amyloidosis and PCL treatment paradigms, unique challenges, and opportunities for further study with novel therapies.

## 2 AL amyloidosis

### 2.1 Clinical presentation & epidemiology

Immunoglobulin light chain amyloidosis (AL amyloidosis, AL) accounts for approximately 70% of all systemic amyloidosis (39); still, the incidence in the U.S. is fewer than 4000 cases per year (40, 41). It is predominantly a disease of older adults; the median age at diagnosis is 64 years, and there is a male predominance of 60% (41).

AL amyloidosis originates in the bone marrow as a clonal plasma cell dyscrasia. Still, the clinical presentation, diagnosis, and management are complicated by the extracellular tissue deposition of the misfolded protein fibrils produced by the clonal cells. This protein deposition leads to end-organ dysfunction and failure, which limits treatment options and increases patient risk. Cardiac and renal involvement are the most commonly involved organs, occurring in 70% and 60% of patients, respectively, but other systems may also be affected, including hepatic (20%), peripheral nerves (15-20%), gastrointestinal (GI) tract (10%), lung and soft tissue (39, 42). This diversity in disease involvement complicates symptom presentation, which can delay diagnosis (43). Various non-specific symptoms can occur at presentation, like fatigue and weight loss, neuropathy, hepatomegaly and transaminitis, pseudohypertrophy, and bleeding diathesis.

Despite the challenges, survival outcomes have improved over the last 40 years from 1.4 years (1980-1989) to 4.6 years (2010-2019), although these gains are substantially lower than what has been observed for MM (44). Five-year overall survival (OS) during these time frames improved from 15% to 48%. The one-year mortality rate is still high at 12-30%, which is primarily related to increased age at diagnosis and advanced cardiac AL involvement (45–47). Delays in diagnosis are resulting in advanced disease and irreversible end-organ damage (48). The incorporation of ASCT in AL therapy contributes to mortality in fit, transplant-eligible patients, as the ASCT treatment-related mortality (TRM) is higher in AL (49, 50). Muchtar and colleagues have developed a predictive tool to assess ASCT day-100 TRM in AL based on 1718 patients at nine centers, using clinical factors and statistical analysis (50). Earlier diagnosis, improved disease detection, earlier effective treatment, better supportive care, and intensified, risk-adapted therapies are vital to improving outcomes in AL (51–53).

### 2.2 Staging & response criteria

Staging and response assessment in AL amyloidosis includes both hematologic and organ components (54). The extent of cardiac involvement is the most important prognostic factor in AL amyloidosis, although the characteristics of the plasma cell clone impact long-term outcomes (55). Other factors also contribute, as the number of involved organs, along with hepatic and autonomic involvement, all impact survival (56). The first reliable staging system was presented by the Mayo Clinic in 2004 (Mayo 2004) and stratified patients into three stages based on troponin-T and N-terminal probrain natriuretic peptide (NT-proBNP) (57). In 2012, Mayo updated the staging model to include both cardiac and hematologic disease parameters (55). Cardiac parameters were adjusted to increase NT-proBNP to 1,800 ng/L and decrease troponin T to > 0.025 ng/mL. The difference between the involved and uninvolved serum free light chains (dFLC) was added to assess the hematologic disease burden. The stages were assigned as I, II, III, and IV based on the patient having 0, 1, 2, or 3 parameters, respectively. In 2015, the European group, to better identify very high-risk patients, proposed amending the Mayo 2004 criteria by dividing the stage 3 patients into 3A and 3B, based on the level of NT-BNP of 8,500 pg/mL (58). More recently, Boston University proposed an update that utilized BNP instead of NT-proBNP or troponin T to allow for accurate staging at facilities with limited access to more specialized testing (59). Staging includes BNP > 81 ng/L or troponin I > 1 ng/mL, stratified as stage I for neither criterion, stage II for one criterion, and stage III for both criteria. Stage III is divided into IIIa and IIIb based on a threshold of BNP > 700 ng/L to better identify very high-risk patients.

Separate response criteria have been developed for hematologic, cardiac, renal, and hepatic involvement. The hematologic response is graded and is based on the absolute difference between the involved and uninvolved serum free light chains (dFLC) (39, 57, 60). Complete response (CR) includes negative serum and urine immunofixation and a normal free light chain (FLC) ratio. Very good partial response (VGPR) is defined as dFLC < 4.0 mg/dL. Partial response (PR) is a 50% reduction in the dFLC. No response is anything less than a partial response.

Organ response criteria have historically been binary, either response or no response and correlate with OS outcomes (61). Cardiac response is based on a decrease in NT-proBNP by > 30% and 300 ng/L. The renal response is based on a 30% reduction in proteinuria or a decrease below 0.5 g/24 hours (h) in the absence of renal progression, which is defined as a >25% decrease in eGFR (48, 62). The hepatic response is defined as a 50% decrease in abnormal alkaline phosphatase or a decrease in radiographic liver size by at least 2 cm (56, 60, 61). A more graded system was proposed by Muchtar et al. in 2019 that mirrored the typical treatment responses seen elsewhere: complete organ response, very good partial organ response, partial organ response, and no organ response, although this has not been universally applied (62).

Minimal (or measurable) residual disease (MRD) plays a significant role in assessing disease response in many hematologic malignancies and may also be associated with survival in AL amyloidosis (63). Several prospective and retrospective small studies have shown a prolonged PFS in those patients achieving MRD negativity (measured by next-generation flow cytometry, level of detection 10^-5^ and 10^-6^), higher MRD negative rates with ASCT than with chemotherapy alone, and an increased likelihood of cardiac response in MRD negative patients (63, 64). One explanation is that low-level continuous amyloid light chain production may contribute to poor organ function over time. A better quantitative assessment of the residual underlying clone may be valuable in this relatively low-cell-burden disease. For example, MRD could help guide treatment decisions for those patients not reaching organ response despite a complete hematologic response. A study by Palladini et al. investigated MRD by next-generation flow cytometry (NGF) in 92 patients with AL amyloidosis in CR (65). Undetectable MRD by NGF was associated with renal organ response in 90% vs. 75% in MRD-positive patients and cardiac organ response in 95% vs. 75%. Hematologic progression was also higher in the MRD-positive group: 25% vs. 0% at 1 year. Further investigation of MRD as a response criterion is needed to understand the full utility of MRD in this group.

Of note, although the cytogenetic changes are similar in MM and AL amyloidosis, these changes have a slightly different impact on outcomes and risk in AL amyloidosis, which is not entirely understood. Translocation t(11;14) is present in up to 61% of AL amyloidosis patients and has historically had a negative impact on PFS due in part to a lower response rate of these patients to bortezomib-based therapies (48, 66, 67). However, this group has responded more favorably to alkylator therapy and ASCT, as well as daratumumab-based therapy (68). Earlier inclusion of frontline anti-CD38 monoclonal antibodies, as well as early assessment of transplant-eligibility and incorporation of bcl-2 inhibitors with preferential activity in patients with t(11;14) may change the impact of this group on overall AL patient outcomes (69).

### 2.3 Treatment approach for newly diagnosed AL amyloidosis

Treatment strategies are primarily directed at eliminating the underlying plasma cell clone to stop fibril production and limit end-organ damage. The goal of therapy is both a rapid and deep response, ideally a hematologic complete response (hCR). Patients with AL amyloidosis are typically more clinically fragile due to end-organ damage, which may limit the feasibility of aggressive myeloma-type regimens or therapies with overlapping toxicities. Early diagnosis, assessment of patient fitness, accurate staging, risk stratification, and appropriate supportive care are paramount when determining a treatment approach (36).

A significant advance in AL amyloidosis has been the successful incorporation of daratumumab with a combination therapy of cyclophosphamide, bortezomib, and dexamethasone (CyBorD) in patients with newly diagnosed (ND) AL amyloidosis (70). The phase 3 ANDROMEDA study compared the combination of daratumumab with CyBorD (dara-CyBorD) with CyBorD alone for six cycles, followed by single-agent daratumumab every 4 weeks for up to 24 cycles. Hematologic CR rate was significantly higher in the daratumumab group (53.3% vs. 18.1% in the control group), as was survival free from major organ deterioration, hematologic progression, or death (70). Later analysis at a median follow-up of 61.4 months found a statistically significant benefit for dara-CyBorD over CyBorD (OS HR 0.62, 95% CI 0.42-0.90, p=0.0121) with the 5-year OS rate of 76.1% for Dara-CyBorD compared to 64.7% for CyBorD. The timing of daratumumab is significant: this 5-year survival benefit of daratumumab upfront in combination was evident even with the vast majority (71%) of patients in the standard CyBorD arm receiving daratumumab later (71). The success of the ANDROMEDA study led to the FDA approval of daratumumab for AL amyloidosis in 2021 (72).

An alternative treatment approach to directly target the misfolded fibrils has gained interest in recent years. The monoclonal IgG1 antibody anselamimab (CAEL-101) binds misfolded immunoglobulin light chains, then promotes phagocytosis and clearance of amyloid deposits (73). Randomized double-blind phase 3 clinical trials of anselamimab combined with CyBorD vs. placebo with CyBorD in newly diagnosed Mayo stage IIIa (NCT04512235) and IIIb (NCT04504825) are ongoing. Birtamimab (NEOD001) is another monoclonal antibody targeting light chains to neutralize and deplete amyloid deposits (74–76). The compound was tested in the VITAL trial, a phase 3 randomized, double-blind trial of birtamimab with bortezomib-based SOC chemotherapy in newly diagnosed AL patients but was stopped prematurely after futility analysis. However, a *post-hoc* analysis suggested a possible benefit of birtamimab for stage IV patients, prompting the AFFIRM-AL study of birtamimab to placebo + SOC therapy in stage IV patients (NCT04973137) (74, 77). A combination of anti-production and promotion of fibril removal may ultimately be needed. A phase 2 study of anselamimab in combination with CyBorD +/- daratumumab is currently ongoing (NCT04304144).

#### 2.3.1 Treatment guidelines

Several organizations have provided guidance and recommendations for managing the complexities of this challenging disease. A comparison of the primary points of therapy is shown in **Supplementary Figure 1**. The consensus guidelines from the National Comprehensive Cancer Network^®^ (NCCN ^®^), Mayo Stratification of Myeloma and Risk-Adapted Therapy (mSMART), the American Society of Clinical Oncology (ASCO), the European Hematology Association (EHA), and the International Society of Amyloidosis (ISA) collectively recommend that all patients with AL amyloidosis be treated as part of a clinical trial at all stages, including initial diagnosis, ASCT, maintenance, and relapsed/refractory disease (39, 56, 78, 79). The goal of therapy, while clearly patient-specific and individually assessed, can be more objectively defined as hCR (per NCCN, EHA, and ISA) or hematologic very good partial response (hVGPR) (per mSMART). Patients should be treated with induction chemotherapy immediately to limit end-organ damage. Those patients with Mayo 2004 stage I-IIIa disease should receive induction chemotherapy with dara-CyBorD. Therapy for stage IIIb patients should be dose-modified dara-CyBorD if available; otherwise, CyBorD or bortezomib, melphalan, and dexamethasone (BMDex) may be alternatives. The recommended duration of induction therapy varies from 2-4 cycles to 6-8 cycles, mainly depending on transplant eligibility status. It is generally advised to continue for two cycles past the best response, as tolerated, to allow for the best disease response possible.

Consensus guidelines also agree that patients should be assessed for transplant eligibility early in therapy and that ASCT should be used carefully in AL patients due to the impact of end-organ dysfunction on TRM (39, 56). Historically, the recommendation has been for patients to proceed to ASCT after 2-4 cycles of induction, and those patients initially transplant-ineligible should be reassessed after induction as they may have become eligible with therapy. However, for those with excellent disease response to induction, ASCT may be deferred. Both NCCN and EHA-ISA guidelines recommend deferring ASCT in those patients with hCR to induction; ASCO guidance supports a threshold of hVGPR. Those deferring ASCT should go on to complete six cycles of Dara-CyBorD followed by eighteen cycles of daratumumab monotherapy as per the ANDROMEDA study (39, 78–80).

Although the consensus guidelines generally agree that there is insufficient data to recommend post-ASCT consolidation or maintenance therapy, most consider a few situations where maintenance may be helpful (39, 56, 78, 79). The mSMART guidelines support maintenance therapy for those with concomitant myeloma or high-risk cytogenetics, acknowledging the limited supportive data and providing the recommendation based on expert opinion only (56). EHA-ISA guidance adds that there may be a role for maintenance in patients with concurrent MM. Several clinical trials are underway investigating the question of maintenance therapy in various settings.

#### 2.3.2 Transplant-ineligible AL amyloidosis

Although ASCT has proven to be important in achieving a significant disease response in AL amyloidosis, only 20% of newly diagnosed patients are transplant eligible (78, 80). The consensus guidelines recommend daratumumab-CyBorD for transplant-ineligible (TIE) patients, with eligibility reassessment after induction. In the absence of access to daratumumab, alternative therapies include a bortezomib-based triplet regimen, such as CyBorD or bortezomib, melphalan, and dexamethasone (BMDex) (56, 78). Based on the ANDROMEDA study and several other retrospective studies showing a hematologic response of 60-80% and CR of 20-25%, bortezomib is a vital component of frontline therapy.

### 2.4 Treatment approach for relapsed/refractory AL amyloidosis

Treatment in the relapsed/refractory setting becomes more complex due to the impact of amyloid fibril deposition on end-organ function. Nearly half of patients do not achieve a CR with daratumumab-based regimens, and only about half (55%) of those who fail daratumumab will go on to have an adequate hematologic response of ≥ VGPR (70, 81). The optimal treatment sequence for 2^nd^ line and beyond is less well-defined. Treatment should be personalized based on patient fitness, personal preferences, degree of end-organ dysfunction, prior classes of therapies received, and other clinically relevant factors (39, 78). Retreatment with the initial therapy is reasonable, particularly if relapse-free for over 2 years (39, 79). Salvage ASCT is also an option for young, eligible, transplant-naïve patients (39). Venetoclax should be considered for patients with t(11;14) (69).

The mSMART relapsed/refractory algorithm provides more specific guidance, suggesting daratumumab as 2^nd^ line for dara-sensitive or naïve patients. For Dara-refractory disease, CyBorD (VCd) is preferred as 2^nd^ line if bortezomib-sensitive; pomalidomide-dexamethasone (Pd) or lenalidomide-dexamethasone (Rd) if bortezomib refractory. Third-line therapy includes ASCT, carfilzomib (for patients without cardiac involvement), venetoclax (for those with t(11;14)), and bendamustine (56). The BCMAxCD3-directed bispecific antibody teclistamab has been attempted in patients with refractory AL amyloidosis and has shown high rates of deep response with low-grade CRS but high rates of infections (82, 83).

Another practical question arises during therapy: At what point should treatment be modified if not PD? The guidelines vary in their advice, but they generally encourage an early move to salvage therapy for less than PR by cycle 2 of induction or < VGPR by cycle 4. Specifically, NCCN advises treatment modification for < PR after cycle two or < VGPR by cycle 3. EHA-ISA has similar guidance: ≤ PR by Cycle 2 or < VGPR by Cycle 3 and no organ response. mSMART guidelines also strongly encourage moving to salvage therapy for those not achieving PR within two cycles or VGPR within four cycles of induction or after ASCT, based on the significant difference in outcomes for patients with CR or VGPR compared to < VGPR (56, 84).

### 2.5 Clinical trial landscape in AL amyloidosis

A comprehensive search of investigational studies on clinicaltrials.gov using the search criteria “AL amyloidosis, amyloid, from dates 1/1/2014-9/17/2024” was conducted, and the results are shown in **Supplementary Table 1A**. Those therapeutic clinical trials identified with the same parameters but with status noted as completed, terminated, withdrawn, or unknown during this period are listed in **Supplementary Table 1B**.

Ongoing clinical trials investigate combinations of daratumumab with various anti-plasma cell regimens, focusing on patients with newly diagnosed (ND) and relapsed/refractory (RR) AL amyloidosis. A key area of interest is the role of ASCT in multi-drug regimens. For ND patients, studies are evaluating ASCT in combination with three- or four-drug regimens, including Dara-CyBorD plus ASCT in a Phase 3 trial (NCT06022939) and Dara-Pom-Dex with ASCT in an investigator-initiated trial (NCT06376214). Additionally, there is a trend toward incorporating targeted therapies in AL amyloidosis, particularly in combination regimens, with multiple Phase 1 and 2 trials exploring venetoclax in both ND and RR settings.

Various advanced therapeutic approaches are under investigation in the RR setting, including antibody-drug conjugates (ADCs), BCL-2 inhibitors, and BCMA-targeted therapies. BCMA-directed therapies encompass T-cell engagers (TCEs), chimeric antigen receptor (CAR) T-cells, and a range of monoclonal, bispecific, and tri-specific antibodies, highlighting the expanding scope of clinical trial options for these patients.

### 2.6 Current CAR T-cell experience in AL amyloidosis

BCMA may be a viable target in AL amyloidosis. In a study by Bal S. et al., patients with AL amyloidosis at Memorial Sloan Kettering Cancer Center from 2012 to 2018 were assessed for BCMA, GPRC5D, and BCL2 expression in bone marrow amyloidogenic plasma cells (52). Among the 27 patients studied, 27 diagnostic and five relapse specimens were evaluated. Median BCMA expression in clonal plasma cells was 80% (range 50-100%) in 25 samples, with GPRC5D at 80% (range 30-100%) in 18 samples and BCL2 expression observed in 92% of samples with the t(11;14) translocation.

A separate study analyzed plasma cell dyscrasia (PCD) patients diagnosed between 2018 and 2021, including 377 patients with BCMA flow cytometry data: 334 with multiple myeloma (MM), 21 with AL amyloidosis, 14 with monoclonal gammopathy of unknown significance, 5 with POEMS syndrome, and 3 with monoclonal gammopathy of renal significance (85). Non-MM patients did exhibit BCMA expression, but to a lesser degree than MM and with greater variability, measured by mean fluorescence intensity. There was no significant difference in BCMA expression among the non-MM diagnoses, possibly due to the small sample size. Non-MM patients also had a lower clonal plasma cell burden, which may be a result of decreased BCMA expression and subsequent loss of the survival advantage BCMA provides (86).

The clinical experience of CAR T-cell therapy in AL amyloidosis has been reported in four case series and one prospective cohort study, which included patients with advanced cardiac and renal disease (**Table 1**). Lebel et al. have reported on the largest cohort of AL patients treated with CAR T (45, 87–89). Sixteen patients with RR AL were included, with a median of four prior lines of therapy (range 3-10), the majority triple-refractory (14/16), and six also resistant to anti-BCMA ADC belantamab mafodotin (87–89). Thirteen had cardiac involvement, five with Mayo stages IIIa or IIIb, and six with NYHA stage III/IV at study entry. All patients received HBI0101 (NXC-201), an anti-BCMA CAR T-cell therapy. The overall hematologic response rate was 94%, with twelve achieving CR (75%), two VGPR, and one PR; MRD negativity was achieved in nine of 14 evaluable patients. Eight of thirteen patients (62%) met organ response criteria, including 78% of those with cardiac involvement. With a median follow-up of 8.4 months (4-31.5 months), the median EFS was 9.6 months (3.3-not reached (NR)), and the median DOR was 8 months (2-NR). Median OS was 10.1 months (5.8-NR). Despite the promising organ responses among cardiac patients, five patients died of cardiac disease: four died of advanced cardiac AL amyloidosis following disease progression, and one died of AL amyloidosis-related cardiac disease. Other toxicities included early hematologic events (prior to day +28), AL-related organ events, and cytokine release syndrome (CRS) without ICANS or treatment-related deaths (**Table 2**). Of the 14 patients that developed CRS, the majority were low-grade: 11 grade 1-2 and 3 grade 3. Hematologic events were higher grade, but most were resolved by day +28. All patients developed grade 4 lymphopenia and hypogammaglobulinemia < 600 mg/dL. Early (< day 28) infections were frequent (56%, 9/16); six were grade 3, two were grade 1-2 respiratory infections, and one was early cytomegalovirus (CMV) reactivation. Late infections included febrile neutropenia in 5/16, three cases of grade 3 pneumonia, one grade 3 COVID-19 infection, and one grade 5 COVID-19 infection.

**Table 1:** Summary of CAR T-cell experience in AL amyloidosis (AL).

Product	N	Median Prior LoT (range)	Prior BCMA	Staging (Organ Disease)	CAR-T cell dose	ORR Heme, Organ	Responses	Median Follow-up (mo)
HBI0101 (87–89)	16	4 (3-10)	N=5	Cardiac n=13, 5 stage IIIa/b	800 × 10^6^ cells (n=13) (range 570 – 1050 x 10^6^ cells)	94%, 62%	CR (n=12) VGPR (n=2) PR (n=1) NR (n=1) MRD neg 9/14	8.4 (4-31.5)
Ide-cel, cilta-cel (35)	6, 2	8 (6-11)	N=3	Stage I n=1, Stage II n=3, N/A n=4 (cardiac n=2, renal n=1, GI n=1, soft tissue n=4)	Unk	62.5%, NE	CR (n=3) VGPR (n=2) NE (n=3)	11 (5.6-26.4)
Ide-cel (90)	1	8	No	Stage II (cardiac, renal)	4.46 × 10^6^ cells/kg	-	VGPR, MRD neg. Renal stable, Cardiac	8.6
Cilta-cel (90)	1	4	No	Stage IV (cardiac)	0.75 × 10^6^ cells/kg	–	sCR, MRD neg. Cardiac response	9
ARI0002h (91)	1	2	No	Stage II (renal)	3 × 10^6^ cells/kg *^a^*	-	sCR, MRD neg. Renal response	12
Anti-CD19 CAR T *^b^* (34)	1	2	No	Stage II renal; Stage IIIa cardiac	Unk	–	VGPR (180 days)	6.5

*^a^* CAR-T cells infused per fractionated protocol on days 0, + 2, + 6 (91).

*^b^* Diagnosis of immunoglobulin M type of AL amyloidosis concurrent with marginal zone lymphoma.

AL, AL amyloidosis; BCMA, B-cell maturation antigen; CAR-T, chimeric antigen receptor T-cell; Cilta-cel, ciltacabtagene autoleucel; CR, complete response; GI, gastrointestinal; Ide-cel, idecabtagene vicleucel; LoT, lines of therapy; mo, months; MRD, minimal residual disease; N/A, not available; neg, negative; NE, not evaluable; NR, no response; ORR, Overall response rate; PR, partial response; sCR, stringent complete response; Unk, Unknown result or not reported; VGPR, very good partial response.

**Table 2:** Cumulative reported adverse events in AL amyloidosis patients treated with CAR T-cell therapy (34, 35, 87–91).

Adverse Event	Grade	Patients (%total) N=28 (100%) ^a^
CAR T-cell Therapy-Related Events		
CRS	Any 3 1-2	22 (79%) 4 (14%) 18 (64%)
ICANS	Any 1	2 (7%) 2 (7%)
Hematologic Events		
Neutropenia	Any 3-4 1-2	19 (68%) 16 (57%) 2 (7%)
Anemia	Any 3-4 1-2	10 (36%) 5 (18%) 3 (11%)
Thrombocytopenia	Any 3-4 1-2	4 (14%) 2 (7%) 2 (7%)
Non-Hematologic Events		
AL Organ-Related Events		
Renal		
Acute kidney injury	Any 2 1	4 (14%) 1 (4%) 3 (11%)
Cardiac		
Cardiac event Acute cardiac failure Cardiac disease	Any 3 5	8 (29%) 3 (11%) 5 (18%)
Liver		
Acute liver injury	Any 1-2 3	6 (21%) 2 (7%) 4 (14%)
Infections Febrile neutropenia Pneumonia	Any Any Any 3	17 (61%) 6 (21%) 4 (14%) 3 (11%)
Respiratory infection	1-2 3	5 (18%) 2 (7%)
BK virus hemorrhagic cystitis CMV reactivation (without disease) H. influenzae sepsis SARS-CoV-2 infection	3 Unk 5 Any 5 3	1 (4%) 1 (4%) 1 (4%) 3 (11%) 1 (4%) 2 (7%)
Worsening depression	Any	1 (4%)

*^a^* A total of 28 AL amyloidosis patients treated with CAR T-cell therapy reported safety data in published literature.

AL, AL amyloidosis; BK virus, human polyomavirus 1; CAR, chimeric antigen receptor; CRS, cytokine release syndrome; CMV, cytomegalovirus; H. influenzae, Haemophilus influenzae; ICANS, immune effector cell-associated neurotoxicity syndrome; MDS, myelodysplastic syndrome; SARS-CoV-2, severe acute respiratory syndrome coronavirus 2; Unk, unknown.

A retrospective study of eight patients with concurrent RR MM and AL amyloidosis showed favorable disease response and safety outcomes (35). Six received ide-cel, and two received cilta-cel. These heavily pretreated patients had a median of eight prior lines of therapy (range 6-11); six (75%) had prior ASCT; all were daratumumab-refractory, and seven were triple-refractory. Three had prior BCMA-directed therapy. This cohort had generally favorable clinical features, with only one high-risk cytogenetic profile (del 17p, gain 1q), four with lower-stage AL, and limited cardiac (two patients) and renal (one patient) involvement. Seven had ECOG performance status ≤ 2. Post-CAR T-cell infusion, three achieved hCR, two hVGPR, and three were unevaluable due to lack of measurable dFLC at infusion. AL responses were rapid, with a median time to best hematologic response of 43 days (range 20-46 days). The two patients with cardiac involvement and one with renal involvement could not be assessed for organ response. Adverse events included CRS (six patients, all ≤ grade 2), ICANS grade 1 (one patient), neutropenia, anemia, thrombocytopenia, and respiratory viral infections.

Four additional cases of CAR T-cell therapy in AL amyloidosis have been reported, three using anti-BCMA CAR T and one with anti-CD19 CAR T (90, 91). The first case involved a woman in her early 60s with relapsed multiple myeloma (MM) and systemic AL amyloidosis with renal involvement, treated with the anti-BCMA CAR ARI0002h in a fractionated dose. She experienced grade 1 CRS, severe neutropenia, and SARS-CoV-2 pneumonia but achieved hematologic partial response (PR) at one month, followed by stringent complete response (sCR) and renal response by 12 months.

The second and third cases were reported by Das et al. in 2023, involving patients with advanced cardiac and renal involvement (90). One patient, a 62-year-old woman with penta-refractory MM and AL amyloidosis, was treated with ide-cel, resulting in VGPR by day +30 and organ response by +9 months, without severe CRS or neurotoxicity. The other, a 33-year-old man with NYHA Class II heart failure and AL amyloidosis received cilta-cel, experienced grade 3 CRS, and achieved stringent complete response with a cardiac response by +9 months. The fourth case involved a 71-year-old man with IgM AL amyloidosis and marginal zone lymphoma, treated with anti-CD19 CAR HD-CAR-1 as 3^rd^ line therapy (34). Despite achieving VGPR by six months, shortly after the six-month visit, he developed a severe respiratory infection due to *Haemophilus influenzae* and subsequent sepsis. He developed multisystem organ failure and died on day +195 after CAR T cell infusion.

The safety profile of CAR T therapy has been a significant concern and potentially limiting factor in discussing CAR T in AL amyloidosis. **Table 2** summarizes overall safety among AL amyloidosis patients reported to date as treated with CAR T-cell therapy. CRS was the most frequently reported CAR T-cell therapy-related event, occurring in 79% of patients, with 64% experiencing grades 1-2 and 15% grades 3-4. ICANS was observed in 7% (n = 2) of patients; one case was grade 1, and for the other case, the grade was not reported. AL organ-related adverse events across all studies included renal involvement in 15% of cases (acute kidney injury, 3 cases grade 1, 1 case grade 2); cardiac involvement in 29% (acute cardiac failure, 3 cases grade 3, cardiac disease 5 cases grade 5); liver involvement in 21% (hepatic dysfunction 6 cases, 2 cases grade 1-2, 4 cases grade 3) (**Table 2**; **Supplementary Table 3A**).

Hematologic toxicities were common, with neutropenia in 76% of patients (52% in grades 3-4), anemia in 38% (19% in grade 2-3, 5% in grade 1), and thrombocytopenia in 24% (10% in grade 2, 5% in grade 3, and one case of grade 4 worsening of pre-existing thrombocytopenia). Non-hematologic events included viral infections: BK virus hemorrhagic cystitis, SARS-CoV-2 pneumonia, severe respiratory infection, respiratory viral infection, and sepsis, each affecting 5-14% of patients.

There does appear to be increased risk after CAR T for those high-risk patients with cardiac amyloid. The experience of CAR T in AL from Israel has been reported previously and has now been updated to include results for 16 patients treated with HBI0101 (45, 87–89). Aggressive supportive care was provided, and cardiac stage IIIb and IV were admitted for cardiac intensive care unit monitoring during and after CAR T infusion. There were no treatment-related deaths, suggesting that these measures were worthwhile and beneficial in the short term. However, even though most patients with cardiac disease showed organ response (7/9 evaluable, 78%), five patients died of cardiac disease within 1 year after CAR T. Four patients had PD with advanced cardiac AL amyloidosis, three of which had achieved a cardiac response after CAR-T therapy, and one died from AL amyloidosis-related cardiac disease.

As of this publication, only two Phase 1 CAR T-cell trials are ongoing for AL amyloidosis (**Supplementary Table 1A**). FKC288 (NCT05978661) is an anti-BCMA CAR T-cell therapy being evaluated in AL amyloidosis and autoimmune kidney disease. NXC-201 (NCT06097832), as previously discussed, is under investigation in the relapsed/refractory AL setting. Neither trial has reported results.

Despite the limited number of cases, these early CAR T reports provide essential insights for AL amyloidosis patients, who were previously considered less tolerant of novel therapy. The findings suggest that targeted anti-plasma cell immunotherapy could hold significant potential for this population, even among heavily pre-treated patients and those with advanced organ dysfunction. With refined supportive care and an evolving understanding of CAR T-cell therapy, the treatment possibilities for AL amyloidosis patients appear increasingly viable and promising (36, 39, 78, 92, 93).

## 3 Plasma cell leukemia

### 3.1 Diagnosis & epidemiology

Plasma cell leukemia (PCL) remains a clear area of unmet need. This very rare and highly aggressive plasma cell malignancy still has poor outcomes despite aggressive multi-modality treatment strategies and the incorporation of novel agents (94). Overall survival ranges from 4 to 12 months; those undergoing ASCT may survive 2 to 3 years (95–97).

PCL comprises approximately 2-4% of all plasma cell dyscrasias and is classified into primary (pPCL), those cases arising *de novo* without a prior diagnosis of MM, and secondary (sPCL), those occurring as leukemic transformation in the setting of MM (98). Historically, the diagnostic criteria for PCL required both circulating plasma cells (CPC) of 20% and an absolute plasma cell count of ≥ 2 × 10^9^/L (99). In 2021, IMWG revised this to the current diagnostic definition of ≥ 5% CPCs in peripheral blood in patients otherwise diagnosed as symptomatic MM. This was based on studies from the Catalan Myeloma Group in Spain and the Mayo Clinic series, showing similarly poor outcomes for patients with 5 to 20% peripheral blood PCs as those with more than 20% (100–102).

When seen concurrently with MM, PCL is an independent predictor of early relapse or progression (103). Patients typically present at a younger median age (52-65 years) than MM but with a higher tumor burden, higher plasma cell proliferation indices, and more bone marrow involvement (98, 104). Consistent with the aggressive nature of the disease, PCL patients are more likely to present with more cytopenias, hypercalcemia, renal failure, a higher beta-2 microglobulin, higher lactate dehydrogenase, and lower albumin at diagnosis (98, 105).

The high rate of high-risk cytogenetic abnormalities in PCL is also thought to be a contributing factor to their poor outcomes, in particular, chromosome 1 abnormalities, del(17p), t(11;14), t(14;16), and high-risk cytogenetic anomalies (97, 106). Primary PCL frequently has changes such as complex karyotypes, hypodiploidy, amp1q, and TP53 mutations, including double-hit profiles and TP53 bi-allelic inactivation, which are also increased in the subset of pPCL patients with t(11;14) (107). These TP53 mutations are associated with significantly lower PFS (4 months vs. 11 months) and OS (5 months vs. 15 months) compared to pPCL patients collectively (107).

### 3.2 Treatment of newly diagnosed pPCL

Because of the aggressive nature of PCL, immediate treatment is advised to decrease tumor burden (104, 108). The approach for PCL therapy is similar to that of high-risk MM with multi-agent induction chemotherapy, including a combination of a PI and an IMiD, followed by stem cell transplant (SCT) for eligible patients, frequently followed by consolidation and/or maintenance regimens (98). The rationale is primarily based on retrospective studies, as most prospective studies of similar regimens in MM excluded PCL patients.

Demonstrating the effectiveness of bortezomib-based regimens (BBR) in PCL was a significant advance in the field (109). A retrospective study of 42 consecutive PCL patients showed a significantly higher overall response (OR) (considered as ≥ PR) with BBR compared with conventional therapies (69% vs. 30.8%, p=0.04) (109). Median OS was significantly improved with BBRs to 13 months vs 2 months, with manageable toxicity. In another retrospective study of 12 patients with PCL, bortezomib both alone and in combination showed improvement in ORR to 92% and responses ≥ VGPR to 50%, with median PFS of 8 months and OS of 12 months, the best responses seen in PCL at that time to date (98, 110). A third retrospective study from the Gruppo Italiano Malattie Ematologiche dell’Adulto (GIMEMA) of 29 newly diagnosed PCL patients treated with bortezomib-containing combination chemotherapy regimens showed an ORR of 79% at a median follow-up of 24 months, and ≥ VGPR in 38% (111). Moreover, 12 of the 29 patients in this analysis successfully received SCT, and those patients had the best outcomes.

One of the only trials dedicated to PCL was the EMN12/HOVON-129 study, which investigated carfilzomib, lenalidomide, and dexamethasone with or without autologous (and/or allogeneic) stem cell transplant for patients with PCL (112). The mPFS was only 15.5 months for younger patients who received transplants; for older patients who did not, the mPFS was only 13.8 months.

The monoclonal antibody daratumumab has also been shown to improve OS and PFS, both in pPCL and sPCL, to 21 months and 20 months, respectively (113). In a retrospective study of patients treated from 2001-2021, 90% were treated with bortezomib-containing regimens and 37% with daratumumab-based regimens. Those treated with daratumumab-based quadruplets or VRD had a significantly longer OS (OS not reached at a median follow-up of 51 months vs. 20 months) and PFS (25 vs. 12 months) (114). The promising role of daratumumab in PCL has translated into several clinical trials that are currently ongoing, primarily investigating the impact of the combination of Dara-VRD in newly diagnosed pPCL with ASCT, either single or tandem, consolidation and maintenance (see **Supplementary Table 2**). The OPTIMUM MUKnine trial included a total of 138 patients with ultra-high-risk cytogenetics, 129 of whom had a diagnosis of MM and 9 with PCL (defined as circulating plasmablasts > 20%). Dara-Cy-VRd induction was followed by ASCT, Dara-VR maintenance, and then Dara-R maintenance (115). The investigators found that such an approach yielded a 30-month PFS of 77%. In the SWOG S1211 study investigating elotuzumab-VRd vs. VRd in high-risk disease, a small number of patients with pPCL were included, and the mPFS was 29 months (116).

#### 3.2.1 HSCT in pPCL

HSCT is still preferred as part of the frontline treatment of PCL based on the aggressiveness of the disease and the success of HSCT in MM, but the ideal approach has not been well-defined (104). Single autologous (auto), single allogeneic (allo), tandem autologous (auto-auto), and tandem autologous followed by allogeneic (auto-allo), with and without maintenance, have all been used (104, 117). A retrospective analysis of the European Society for Blood and Marrow Transplantation (EBMT) experience from 1998-2014 was published by Lawless et al. using dynamic prediction modeling to compare these approaches (118). The study found that for those with pPCL in CR prior to the first auto transplant, tandem auto-auto transplant had similar outcomes as auto-allo, while avoiding the higher non-relapse mortality (NRM) with allo and the risk of graft-versus-host disease (GVHD). For those in less than CR prior to the first auto transplant, auto-allo showed superior OS.

Another retrospective data analysis from the Center for International Bone and Marrow Transplant Research (CIBMTR) studied outcomes of patients with pPCL treated with HSCT from 2008 to 2015 (117). Of the 277 patients in the auto cohort, 90% received single auto, and 10% received tandem auto (auto-auto). Of the 124 patients for which induction chemotherapy was known, 83% received a bortezomib-based regimen (CyBorD or VRD), and 76% received one line of therapy. Only 19% were in CR at the time of transplant and 28% in VGPR; 40% were in PR, 6% stable disease (SD), and 5% progressive disease (PD). Median follow-up was 48 months (range 3-84 months). Planned post-HSCT maintenance therapy was given to 27% of patients in the auto cohort. Sixty-one percent of patients had died at the time of last follow-up, 85% of those from PD. Four-year PFS was 17%; 4-year OS was only 28%. Non-relapse mortality (NRM) was 7%, and the incidence of relapse/progression was 76%. Results from the allogeneic cohort were similarly dismal. Seventy-one patients received an allo HCT for pPCL, of whom 61% were single allo while 39% were tandem auto-allo. Induction therapy was known in 37 of the 71 patients; 86% had received bortezomib-based therapy (VRD or VDPACE), and 70% had received only one prior line of therapy. Disease response at transplant was slightly better; 21% were in CR, 27% VGPR, 34% PR, 7% SD, and 11% PD. A slight majority (55%) of allo patients received a non-myeloablative or reduced-intensity preparative regimen, and 51% received total body irradiation. Planned post-HCT therapy was reported as given in only 12% of patients. The median follow-up for the allo cohort was 60 months (range 6-92 months). At the last follow-up, 63% of the allo cohort had died, 76% from relapsed or progressive disease. Four-year PFS was 19% and 4-year OS 31%, with NRM 12% at 4 years and incidence of relapse/progression 69%. The authors did note an increased utilization of HSCT in this era, attributed to the addition of novel agents and improved induction regimens, and an improvement in NRM with improvement in transplant practice overall. Unfortunately, post-HSCT outcomes remained poor, predominantly because of the high rates of post-HCT relapse.

#### 3.2.2 ASCT in pPCL

Adapting to the current standards of care in myeloma, proposed regimens for fit, transplant-eligible patients would be induction therapy with an anti-CD38 monoclonal antibody, proteasome inhibitor, lenalidomide, and dexamethasone with or without cyclophosphamide, followed by at least one autologous transplant and prolonged maintenance with an anti-CD38, proteasome inhibitor, and lenalidomide. Management of patients not eligible for transplant should balance toxicities with efficacy, allowing patients to receive continuous treatment for as long as possible (104, 108). Transplant-ineligible patients may benefit from prolonged triplet or even quadruplet myeloma therapy. Both transplant-eligible and transplant-ineligible patients may take advantage of the introduction of anti-CD38 monoclonal antibodies in induction therapy. For patients in need of rapid reduction in disease burden at diagnosis of pPCL, more aggressive combinations like VTd/VRd-PACE or hyperCVAD-RV are suitable options (105). CyBorD can be used as a less intensive option for more frail patients who still need rapid disease response (96).

### 3.3 Treatment of sPCL

Unfortunately, outcomes in relapsed or refractory PCL remain very poor, with a median OS of 7 months despite aggressive treatment upfront with multi-drug induction, HSCT, and novel therapies (104). Allogeneic SCT may be beneficial in eligible patients with chemo-sensitive disease. It is critical to utilize drugs active in MM to which the patient has not previously been exposed. However, despite various combinations of conventional plasma cell-directed therapies, sPCL often necessitates the use of aggressive cytoreductive chemotherapy for disease control. T-cell-engaging immunotherapies such as bispecific antibodies and CAR T-cell therapy may represent a new option for patients with sPCL.

### 3.4 Prospective clinical trials landscape in PCL

Currently, clinical trials involving pPCL are addressing the role of SCT, the approach to combination drug regimens, and the question of CAR T-cell therapy across newly diagnosed, transplant-ineligible, and relapsed/refractory settings. A comprehensive search of investigational studies on clinicaltrials.gov using the search criteria “plasma cell leukemia, primary plasma cell leukemia, from dates 1/1/2014-9/17/2024”, excluding those trials not directed at the underlying disease, i.e., supportive care studies, drug formulation studies, etc. The resulting studies are shown in **Supplemental Table 2**.

Most clinical trials available for PCL patients are still designed mainly as MM studies but allow for PCL patients. At the time of this writing, all available clinical trials for RR PCL are for CAR products in RR MM trials, primarily investigator-initiated trials (IIT) and early phase 1 studies, and limited to primary PCL. However, new studies exclusively for pPCL are now becoming available. In the newly diagnosed setting, a phase 2 study investigates daratumumab-PI-IMiD combinations as consolidation with tandem ASCT and lenalidomide maintenance (NCT05054478). An IIT in newly diagnosed pPCL patients includes VRd induction with a “triple tandem” design of anti-BCMA CART-ASCT-CART2 (NCT05870917). Another phase 2 anti-BCMA CAR T study is available for transplant-ineligible patients using VRd induction in combination with CAR T (NCT05979363). With a higher incidence of t(11;14) in PCL, there is enthusiasm for the addition of venetoclax or other bcl-2 inhibitors, although their role is still unclear (33, 48, 107, 119).

### 3.5 CAR T experience in PCL

CAR T-cell therapy for PCL, while scientifically rational, has not been well studied owing in part to concern about higher toxicities and lower efficacy (**Table 3**). The published CAR T-cell experience in PCL to date is shown in **Table 4**. There have been four published reports of CAR T-cell use in a total of 22 RR PCL patients: a multicenter retrospective study of 15 patients with PCL, a retrospective study of 8 patients with sPCL in China, a report of 2 patients included in an MM trial, and another patient treated with CAR T therapy for sPCL after RR MM (32, 33, 120). It is noted that these studies included both pPCL and sPCL, two biologically distinct diseases. Combining this data does create a limitation in the conclusions. However, despite the distinct biological features and outcomes of pPCL and sPCL, there are still lessons to be learned from a collective review of the CAR T experience in these patients.

**Table 3:** Cumulative reported adverse events in plasma cell leukemia (PCL) patients treated with CAR T-cell therapy (32, 33, 120, 121, 132).

Adverse Event	Grade	Patients (%total) N=20 (100%) ^a^
CAR T-cell Therapy-Related Events		
CRS	Any 1-2 3-4 5	19 (95%) 16 (80%) 3 (15%) 0 (0%)
ICANS	1	4 (20%)
Infection Severe pneumonia Pulmonary aspergillosis	Any 5 Unk	8 (40%) 1 (5%) 1 (5%)
Hematologic Events		
Neutropenia	Any 2 3	18 (90%) 1 (5%) 7 (35%)
Anemia	Any 3	16 (80%) 8 (40%)
Thrombocytopenia	Any 3 4	14 (70%) 1 (5%) 7 (35%)
Non-Hematologic Events		
Nausea and Vomiting	1 3	4 (20%) 4 (20%)
GI hemorrhage	5	1 (5%)

*^a^* A total of 20 PCL patients treated with CAR T-cell therapy reported safety data in published literature.

CAR, chimeric antigen receptor; CRS, cytokine release syndrome; GI, gastrointestinal; ICANS, immune effector cell-associated neurotoxicity syndrome; Unk, unknown.

**Table 4:** Summary of CAR T-cell experience in PCL.

Product	N	Median Prior LoT (range)	Prior BCMA	Type	CAR-T cell dose	ORR	Responses	Median Follow-up (mo)
Ide-cel (120)	11	6 (0-15)	Unk	pPCL (n=4) sPCL (n=7)	413 × 10^6^ cells (range 331-455 × 10^6^ cells)	100% (pPCL) 57% (sPCL)	sCR (n=1) VGPR (n=1) PR (n=2) CR (n=1) VGPR (n=2) PR (n=1)	6 (0-15) *^a^*
Anti-BCMA CAR T (121)	8	3.5 (1-7)	Unk	sPCL (n=8)	Unk	75% ORR 62.5% 6mo PFS 60% 6mo OS	VGPR (n=2) PR (n=4)	6.2
PLVX-BCMA-01 (32)	2	4 (3-11) *^b^*	No	pPCL	11.2 × 10^6^ cells/kg *^c^*	-	CR, PFS 307d VGPR, PFS 117d	Unk
Anti-BCMA CAR T (33)	1	5	No	sPCL	0.37 × 10^6^ cells/kg	–	sCR, PFS 16 months	N/A
CT103A (132)	1	4 (3-6) *^c^*	Unk	sPCL	3 × 10^6^ cells/kg	-	VGPR, OS 225d	7.5

*^a^* Median follow-up listed applies to both pPCL and sPCL patients.

*^b^* Median LoT for all participants (MM and PCL), LoT for PCL patients not provided.

*^c^* Median CAR-T dose for study, including MM and PCL patients, range 5.4-25.0 × 10^6^ cells/kg. Dose for PCL patients not provided.

BCMA, B-cell maturation antigen; CAR-T, chimeric antigen receptor T-cell; CR, complete response; d, days; Ide-cel, idecabtagene vicleucel; LoT, lines of therapy; MM, multiple myeloma; mo, months; N/A, not available; neg, negative; NR, no response; ORR, Overall response rate; OS, overall survival; PCL, plasma cell leukemia; PFS, progression-free survival; pPCL, primary plasma cell leukemia; PR, partial response; sCR, stringent complete response; sPCL, secondary plasma cell leukemia; Unk, Unknown result or not reported; VGPR, very good partial response.

Fortuna et al. published the most extensive retrospective anti-BCMA CAR T study in PCL patients (120). Fifteen patients underwent leukapheresis with the intent of receiving CAR T therapy; 11 were infused, and four patients died due to disease-related complications before infusion. The majority of patients in the group had high-risk cytogenetics (73%), including t(14;16), t(4;14), 1q21 gain or amp, and del17p, and 33% had t(11;14). All patients were triple-refractory, and 40% were penta-refractory, with a median of 6 prior lines of therapy (range 4-9). For the 11 patients infused, the median dose of ide-cel was 413 × 10^6^ cells (range 331-455 × 10^6^ cells). The response rate was 100% in the pPCL patients: 1 sCR, 1 VGPR, 2 PR. Of the sPCL patients, 4 of the seven dosed patients responded (57% response rate): 1 CR, 2 VGPR, 1 PR. Unfortunately, the median PFS (mPFS) for the cohort overall was only 3.7 months (2.8-NR months), and the median OS (mOS) was 6.7 months (4.6-NR months). Although sPCL had a significantly shorter mOS, there was no difference in mPFS between the two groups. Survival was longer for pPCL, but still only mOS of 8.1 months vs. 4.6 months. CRS was frequent but primarily low-grade; 81% of patients reported CRS, 11% grade 3, and the remainder grade 1-2. Approximately one-third of patients had ICANS (36%), all grade 1. Other adverse events included infection in 55%, neutropenia in 45%, anemia in 73%, and thrombocytopenia in 54% (grades not reported).

Another retrospective study of anti-BCMA CAR T-cell therapy in 8 patients with sPCL was published by Guo et al. (121). Patients were treated from December 2020 until November 2022 with a median of 3.5 prior lines of therapy (range 1-7, 62.5% greater than five lines). All patients were triple refractory; half were also resistant to pomalidomide. Three patients had been treated with SCT previously. ORR at 1 and 2 months after CAR T-cell therapy was 75%; 4 patients had PR, and 2 had VGPR. Three of the six patients in remission went on to allogeneic SCT 3 months after CAR T; two of those patients are still alive with sCR, although the DOR was not published, and one died shortly after SCT. Of the other three PR patients who did not receive allo-SCT, two died after relapse, and one remains in VGPR follow-up. Similar to the prior reports, CRS and myelosuppression were frequent. All eight patients developed CRS: 4 with Grade 1, 2 with Grade 2, and 2 with Grade 4. There were no cases of ICANS. Other adverse events included neutropenia Grade 2-3 in all patients, anemia Grade 3 in all patients, thrombocytopenia Grade 3-4 in all patients, and nausea/vomiting in all patients. Three patients died due to infectious and bleeding complications. One patient died within one month of CAR T infusion due to severe pneumonia, and another also died within a month of CAR T due to gastrointestinal hemorrhage. Both patients had up to 70% abnormal peripheral blood plasma cells. The third patient died 3 months after CAR T with pulmonary Aspergillus infection.

Zhou et al. published the results of their early phase I study of anti-BCMA CAR T-cell therapy in RR MM, including two patients with pPCL who benefited from therapy. One patient achieved a CR with a PFS of 307 days; the second had a VGPR with a PFS of 117 days (32). Gao and colleagues published their experience with anti-BCMA CAR T-cell therapy in a patient with sPCL in the setting of R/R MM after five prior lines of therapy (33). The patient had a stringent complete response (sCR) for 9 months after CAR T-cell infusion, then venetoclax to maintain a complete remission (CR) for another 7 months. Neither of these reports included specific safety data regarding CRS, ICANS, or other adverse events for those pPCL patients.

Overall, the disease response trend has been promising but short-lived: patients respond to therapy for a limited period compared to MM. Based on the small number of patients, it is unclear if the risk is higher in sPCL than in pPCL.

## 4 Discussion

### 4.1 Challenges of current therapy for plasma cell dyscrasias

**Figure 1:**
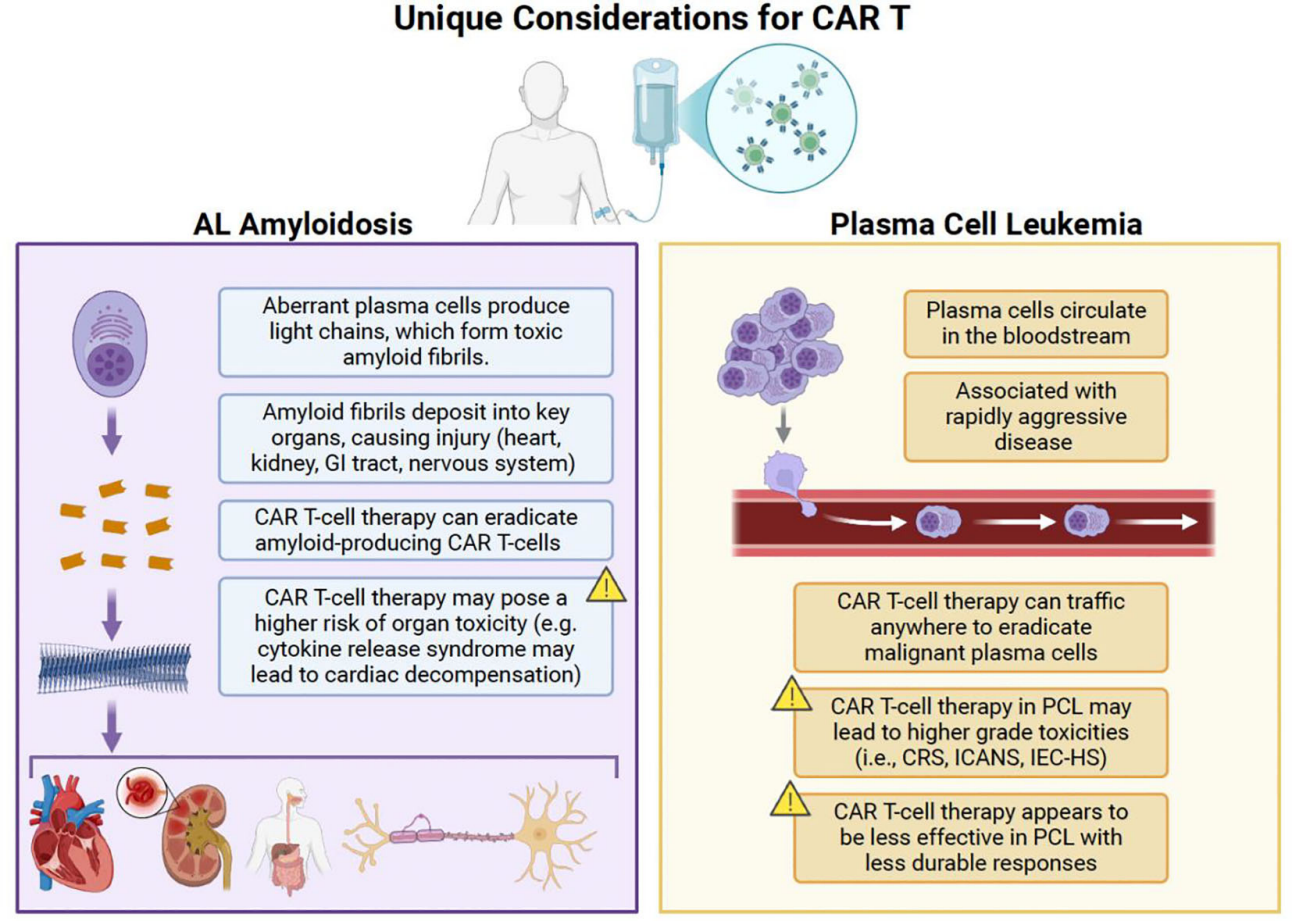
Unique considerations for the use of CAR T in AL amyloidosis and PCL. CAR T, chimeric antigen receptor T-cell therapy; CRS, cytokine release syndrome; GI, gastrointestinal; ICANS, immune effector cell-associated neurotoxicity syndrome; IEC-HS, immune effector cell-associated hemophagocytic lymphohistiocytosis-like syndrome; PCL, plasma cell leukemia. Created with BioRender.com.

Plasma cell dyscrasias are a diverse group of rare diseases. Although the disease trajectory of AL amyloidosis and PCL differ, both diseases can be physiologically damaging to the patient to an extent that can limit therapy options. The unique considerations of CAR T in these indications are shown in **Figure 1**. End organ damage in AL amyloidosis puts patients at significant risk of cardiac, renal, hepatic, and other organ insufficiencies that make it challenging to complete aggressive, prolonged regimens with multiple agents. Rapid tumor growth, aggressive disease presentation, and high disease burden in PCL put patients at risk for tumor lysis syndrome, significant cytopenias, bleeding diatheses, and hypercalcemia (122). Both are similar enough to MM to have borrowed from MM treatment approaches and have had variable amounts of success. However, the prolonged treatment course involving induction, consolidation, and maintenance is challenging for some patients to complete. CAR-T’s single-time point therapy design may be a good alternative to a prolonged regimen with the risk of treatment interruptions, dose reductions, and adverse events.

### 4.2 Cell therapy-specific risks in AL and PCL

Additional disease-specific risks exist in the setting of stem cell mobilization, and leukapheresis demonstrated in the SCT setting for AL amyloidosis, which should also be considered in the context of CAR T. Stem cell mobilization in the SCT setting in AL amyloidosis is associated with increased hypotension, hypoxia, cardiac arrhythmia, and fluid retention, particularly in those AL patients with congestive heart failure or nephrotic syndrome (80). Such significant sequelae can interfere with lymphodepletion conditioning chemotherapy through treatment delays or dose modifications. In the SCT setting, the additional need for granulocyte colony-stimulating factor (G-CSF) increases the risk of volume overload and capillary leak; the lack of G-CSF use in lymphocyte apheresis for CAR T-cell therapy limits this risk.

The dosing of conditioning regimens may also need further consideration. In full-intensity conditioning regimens for SCT in AL amyloidosis, treatment-related mortality (TRM) rates of up to 20% have been noted (80). Accurate patient selection and risk-adapted dose modifications based on age, cardiac, and renal function have substantially reduced TRM to 2-10% (56, 80). These dose modifications are not without their own risk; a modified dose of melphalan (140 mg/m^2^) has been associated with lower CR rates and decreased OS. The lymphodepletion regimen of cyclophosphamide and fludarabine used in CAR T conditioning is tolerated well overall in similar populations of heavily treated RR MM patients. However, the use of fludarabine raises concerns for patients with renal involvement of AL amyloidosis. Induction chemotherapy may be beneficial for improving end-organ function prior to lymphodepletion chemotherapy (56). Conditioning chemotherapy prior to CAR T for the AL patients included in **Table 1** generally included cyclophosphamide 300 mg/m^2^/dose x 3 doses and fludarabine 25-30 mg/m^2^/dose x 3 doses for those with sufficient renal function (creatinine clearance ≥ 30 mL/min or per institutional standards) (35, 87, 88, 90, 91). For those with decreased renal function, fludarabine dosing was reduced, or bendamustine 90 mg/m^2^ was used. The optimal dosing of fludarabine in patients with AL and renal involvement requires further investigation.

By contrast, TRM is not a primary limitation for PCL patients. According to a review from CIBMTR of 348 patients with pPCL treated between 2008 and 2015, non-relapse mortality (NRM) in the transplant setting was 7% for auto-HCT, still higher compared to auto-HCT in MM (123, 124) and 12% for allo-HCT (117). Even in these patients treated with SCT in the modern era of novel agents in the front-line setting, the rate of relapse within 4 years post-SCT was still high at 69-76%.

### 4.3 Updated safety of CAR T-cell therapy

The primary concern regarding the use of CAR T-cell therapy in AL amyloidosis is safety, particularly in patients with cardiac impairment, renal insufficiency, or other forms of end-organ dysfunction. Intrinsic to the success of CAR therapy is the activation of the immune system via cytokines such as IL-1, IL-6, IL-10, TNF-a, and interferon (IFN)-g to kill tumor cells (125, 126). These cytokines are also responsible for vascular leakage and disseminated intravascular coagulation (DIC), which can be life-threatening. Excessive stimulation of T-cells, cytokine production, and cytokine release from macrophages after CAR therapy results in Cytokine Release Syndrome (CRS), a frequent clinical syndrome manifesting in systemic inflammation, increased vascular permeability, and possible neurotoxicity (126). With CAR experience has also come improved management of CRS, including standardized assessment of grade and severity, prompt administration of tocilizumab with or without corticosteroids, management of infections, and multidisciplinary supportive care (19, 20, 126). In a real-world data study from the US Myeloma Immunotherapy Consortium of 159 patients at 11 centers in the USA, the rate of CRS with ide-cel was 82% overall but only 2% in grades 3-4 and 1% in grade 5 (127). In a similar study of real-world data outcomes from cilta-cel, the US Myeloma Immunotherapy Consortium reported on 139 patients treated; the rate of CRS was similar at 81% overall with 7% grade 3 or higher (128). Of those cases of AL treated with CAR T-cell therapy reported here, 15 of the 21 patients (71%) experienced CRS of any grade, with 12 being grades 1-2 and 3 with grade 3 (see **Table 2**). Other cumulative reported safety events for AL patients receiving CAR T therapy are shown per study in **Supplementary Table 3A**. The cumulative safety data for the published PCL cases treated with CAR T-cell therapy are shown in **Table 3**; **Supplementary Table 3B** shows safety data for PCL patients by study. CRS was very frequent but low grade: 19/20 (95%) had any grade of CRS. The majority were grade 1-2 (16/19, 80%). Although common in both AL and PCL, CRS is still predominantly mild, requiring minimal support, even in these vulnerable patients.

The most serious sequelae seen in these groups of patients seem to be infections and hematologic events. As with multiple myeloma (129), unexpected serious adverse events in the PCL patients included three deaths after CAR T, two of which occurred in the first month after CAR infusion, and both in patients with increased circulating plasma cells of up to 70%. One of these deaths was due to severe pneumonia and the other to gastrointestinal hemorrhage. A third patient died 3 months after CAR T with pulmonary Aspergillus infection. Among the AL amyloidosis CAR T patients, one patient died due to severe infection, subsequent sepsis, and multiorgan failure. Despite small numbers, these populations continue to see high-grade adverse events. It is unclear which variables may contribute to these outcomes, but further study should explore ways to improve the safety of cell therapies for these patients.

### 4.4 CAR T-cell efficacy in AL and PCL

Because of the rarity and clinical behavior of AL amyloidosis and PCL, these diseases have historically been excluded from most other clinical trials studying novel therapies, such as CAR T-cell therapy. CAR T is appealing for AL amyloidosis because, as a low-burden disease, there may be a more favorable response and less risk of infections, cytopenias, and CRS, as is seen in low-tumor burden MM (130). Clinical trials for both diseases are underway to study these novel agents and better understand the role of the currently available therapies like SCT, PIs, IMiDs, anti-CD38 antibodies, and others.

**Tables 1** and **4** summarize the published experience of CAR T therapy in AL and PCL to date, respectively. All patients were treated with anti-BCMA CAR T agents, some with commercially available and others with investigational compounds. Stages varied in AL amyloidosis patients; in PCL, both primary and secondary PCL were included. CAR T-cell dose varied widely from 0.37 × 10^6^ cells/kg to 800 × 10^6^ cells, with varying responses. Median follow-up for all patients was at least 6 months (range 6-26.4 mo.). Among 21 AL patients treated with CAR T, 10 had sCR or CR, 6 had VGPR, 1 had PR, and four were not evaluable. Of 23 PCL patients, 5 had pPCL, and 17 had sPCL. Four patients had sCR or CR, 7 had VGPR, and 7 had PR. Unfortunately, the long-term outcomes after CAR T for patients with AL or PCL are well documented owing to short follow-up or a lack of granular details in reports on these patients. Despite high OR rates, the duration of response and overall outcomes still seem suboptimal. However, the experience is promising and deserves more investigation to find a better therapy combination or at least add another therapy option.

Further prospective studies are needed to understand the impact of these responses and post-CAR relapse risk in these groups. Despite the variability in product, dose, and disease demographics, these results are encouraging, suggesting that cell therapies may benefit these complex patients.

In addition to BCMA-directed therapies, other CAR T targets such as GPRC5D and CD229 also appear promising for plasma cell dyscrasias. CD229-directed CAR T was investigated in MM and included 3 PCL patients (131); the CD138+ tumor cells showed high CD229 expression. The anti-CD229 CAR T cell exhibited high cytotoxicity and pro-inflammatory cytokine production against these tumor cells, suggesting it could be a useful target. There are several ongoing GPRC5D-directed CAR T-cell studies in myeloma, and prior studies have already demonstrated high rates of GPRC5D expression in MM and AL amyloid (52).

### 4.5 Study limitations

The study of rare diseases is inherently limited and challenging. Large-scale clinical research on CAR T-cell therapies for AL amyloidosis and PCL remains constrained by the low incidence of these conditions and the exclusion of specific patient populations from prior trials. While the reviewed studies provide valuable insights, they predominantly consist of descriptive case reports or retrospective cohort analyses. The evaluation of outcomes, efficacy, and safety events is further restricted by limited detailed data in the original publications, as individual patient-level data is inaccessible for meta-analyses. Additionally, cross-study comparisons are hindered by the heterogeneity of anti-BCMA CAR T products, including ide-cel, cilta-cel, HBI0101, ARI0002h, CT103A, and other academic CAR T therapies, making it difficult to establish a standardized evaluation of therapeutic experiences across studies.

## 5 Future directions

Although the treatment of plasma cell disorders has advanced with novel therapies, better supportive care, and aggressive regimens, there is still work to do. Relapsed and refractory AL amyloidosis and PCL have ground to gain compared to their ND and MM counterparts. Studies specific to PCL and AL amyloidosis are understandably challenging to conduct due to the rarity and acuity of these diseases. It is imperative that clinical trials allow for the inclusion of these underserved populations; for these rare diseases, having even a small number of patients in a clinical trial is valuable.

CAR T-cell therapies warrant further exploration in the context of AL amyloidosis and PCL. Patient selection should be performed with caution, alongside proactive supportive care, especially for those with known end-organ dysfunction. Enhancing the efficacy of CAR T-cell therapy requires advancements in target specificity, prolonging cellular persistence *in vivo*, and mitigating therapy-related toxicities. Dose modification for either lymphodepletion or CAR T-cell products or both may be helpful to improve safety and reduce adverse events. Early-line use of CAR T-cell therapy may improve efficacy compared to use in heavily pretreated patients and minimize toxicity in patients with significant end-organ impairment. Alternative targets, such as GPRC5D and CD229, present promising avenues for investigation. Furthermore, future studies should incorporate basic science and genomic analyses to deepen our understanding of the pathophysiology underlying these conditions and refine therapeutic strategies.
