# Supplementary material for: Changing lanes: extending CAR T-cell therapy to high-risk plasma cell dyscrasias
Source: Front Immunol. 2025 Apr 8;16:1558275. doi: 10.3389/fimmu.2025.1558275 (PMC12011880; doi:10.3389/fimmu.2025.1558275)
Supplement: Supplementary file 1 [file DataSheet1.zip › Suppl Table 3B Safety Summary by Study PCL.docx]

Supplemental Material Table 3B: Safety event summary for PCL by publication.

| Reference | Diagnosis | N | Adverse Event (N) | Any Grade  N (% N by study) | G1-2 | G3-4 | G5 |
| --- | --- | --- | --- | --- | --- | --- | --- |
| (1) Fortuna et al. | PCL | 11 |  |  |  |  |  |
|  |  |  | CRS | 9 (81%) | 8 | 1 |  |
|  |  |  | ICANS | 4 (36%) | 4 |  |  |
|  |  |  | Hematologic (d +90) *^a^* | Unk |  |  |  |
|  |  |  | Neutropenia | 5 (45%) |  |  |  |
|  |  |  | Anemia | 8 (73%) |  |  |  |
|  |  |  | Thrombocytopenia | 6 (55%) |  |  |  |
|  |  |  | Infection | 6 (55%) |  |  |  |
|  |  |  |  |  |  |  |  |
| (2) Deng et al. | sPCL | 1 |  |  |  |  |  |
|  |  |  | CRS | 1 (100%) | 1 |  |  |
|  |  |  | ICANS | 0 (0%) |  |  |  |
|  |  |  | Infection | 1 (100%) |  | 1 |  |
|  |  |  |  |  |  |  |  |
| (3) Li, C et al. | pPCL | 2 | No safety data provided *^b^* |  |  |  |  |
|  |  |  |  |  |  |  |  |
| (4) Guo et al. | sPCL | 8 |  |  |  |  |  |
|  |  |  | CRS | 8 (100%) | 6 | 2 |  |
|  |  |  | ICANS | 0 (0%) |  |  |  |
|  |  |  | Hematologic | 8 (100%) |  |  |  |
|  |  |  | Neutropenia | 8 (100%) | 1 | 7 |  |
|  |  |  | Anemia | 8 (100%) |  | 8 |  |
|  |  |  | Thrombocytopenia | 8 (100%) |  | 8 |  |
|  |  |  | Infection  Pneumonia  Pulmonary aspergillosis | 2 (25%)  1 (12.5%)  1 (12.5%) |  |  | 1  1 |
|  |  |  | Other  GI hemorrhage | 1 (12.5%)  1 (12.5%) |  |  | 1 |
|  |  |  |  |  |  |  |  |
| (5) Li C et al. | sPCL | 1 | Limited safety data provided *^c^* |  |  |  |  |
|  |  |  | CRS | 1 (100%) | 1 |  |  |
|  |  |  | ICANS | 0 (0%) |  |  |  |

Abbreviations: AL, AL amyloidosis; CHF, congestive heart failure; CRS, cytokine release syndrome; d, day; DVT, deep vein thrombosis; G, grade; GI, gastrointestinal; ICANS, immune effector cell associated neurotoxicity syndrome; N, number of patients; PCL, plasma cell leukemia; pPCL, primary plasma cell leukemia, SARS-CoV2, severe acute respiratory syndrome coronavirus 2; sPCL, secondary plasma cell leukemia; Unk, unknown.

^a^ Authors reported adverse events cumulatively as “any grade”, without providing data on quantities of categorical grades. Cumulative total events reported here with this limitation.

^b^ Authors reported 2 cases of pPCL in context of study of multiple myeloma patients. Safety outcomes and events were not reported in the source data.

^c^ Authors reported 1 case of sPCL in context of study of multiple myeloma patients. Limited safety outcomes were reported specifically for the sPCL patient. No ICANS events were reported for the cohort.

### REFERENCES

1. Fortuna GMG, Sidana S, Hovanky V, Khouri J, Dima D, Kocoglu MH, et al. Idecabtagene Vicleucel (ide-cel) Chimeric Antigen Receptor T-Cell for Plasma Cell Leukemia (PCL): A Multicenter Experience. Transplantation and Cellular Therapy, Official Publication of the American Society for Transplantation and Cellular Therapy. 2024;30(2):S381-S2.

2. Deng J, Lin Y, Zhao D, Tong C, Chang AH, Chen W, et al. Case report: Plasma cell leukemia secondary to multiple myeloma successfully treated with anti-BCMA CAR-T cell therapy. Front Oncol. 2022;12:901266.

3. Li C, Cao W, Que Y, Wang Q, Xiao Y, Gu C, et al. A phase I study of anti-BCMA CAR T cell therapy in relapsed/refractory multiple myeloma and plasma cell leukemia. Clin Transl Med. 2021;11(3):e346.

4. Guo Y, Hu K, Ke X, Yang F, MA L, Xu T, et al. PB2101: A Prospective Investigation Into the Timing and Status of BCMA-CART in Secondary Plasma Cell Leukemia. HemaSphere. 2023;7(S3):e640168c.

5. Li C, Wang Q, Zhu H, Mao X, Wang Y, Zhang Y, et al. T Cells Expressing Anti B-Cell Maturation Antigen Chimeric Antigen Receptors for Plasma Cell Malignancies. Blood. 2018;132(Supplement 1):1013-.
