# Supplementary material for: Changing lanes: extending CAR T-cell therapy to high-risk plasma cell dyscrasias
Source: Front Immunol. 2025 Apr 8;16:1558275. doi: 10.3389/fimmu.2025.1558275 (PMC12011880; doi:10.3389/fimmu.2025.1558275)
Supplement: Supplementary file 1 [file DataSheet1.zip › Supplementary Figure 1, Treatment Guidelines. v1.1.pdf]

Changing Lanes: Extending CAR T-cell Therapy to High-Risk Plasma Cell Dyscrasias

Morgan HT, Derman BA, Ma H, Kumar SK

Supplemental Material Figure 1: Comparison of consensus treatment guidelines for systemic AL amyloidosis.

	NCCN (1)	ASCO (2)	mSMART (3)	EHA-ISA (4, 5)
**Clinical Trials** [a]	Clinical trials are recommended for all patients when possible.			
**Therapy Goal**	hCR: neg FLC by serum IFE & urine IFE; FLC ratio in normal range or uFLC > iFLC	Not provided	≥ hVGPR: dFLC < 40 mg/L	hCR: iFLC < 20 mg/L or dFLC < 10 mg/L
**Induction** [b]	Dara-CyBorD, number of cycles not specified	Dara-CyBorD x 6 cycles	Dara-CyBorD x 2-4 cycles	Dara-CyBorD x 2-4 cycles
**ASCT**	ASCT if eligible after 2-4 cycles of induction.<br><br>May defer ASCT if hCR with induction.	ASCT if eligible after 2-4 cycles of induction.<br><br>May defer ASCT if ≥ hVGPR.	ASCT if eligible after 2-4 cycles of induction.<br><br>May defer ASCT if low disease burden.	ASCT if eligible. Consider deferring ASCT if hCR after 2-4 cycles of [illegible]
**ASCT Ineligible**	Dara-CyBorD induction, reassess ASCT eligibility after therapy response.			
**Maintenance**	Not routinely recommended.	Dara x 2 years	Consider for MM phenotype or high-risk FISH abnormalities.	Not routinely recommended unless concurrent MM.
**Treatment Modification** [c]	< PR after Cycle 2<br>< VGPR after Cycle 3	If PR, add 2 more cycles of induction, then ASCT.	If < PR within 2 cycles or VGPR within 4 cycles or after ASCT.	≤ PR by Cycle 2<br>< VGPR by Cycle 3 + no OR
**Relapse / Refractory**	Consider repeating initial therapy, especially if relapse-free for several years.<br><br>Multiple combination options of PI + IMiD + Dex, CYC, Mel/Dex, Bendamustine + Dex, Venetoclax ± Dex.	Consider repeating initial therapy off-therapy ≥ 2 yrs.<br><br>• Dara-based doublet or triplet regimen.<br>• V-based or IMiD-based.<br>• Venetoclax if t(11:14)<br><br>Consider ASCT.	• Dara-based therapy<br>• CyBorD, Mel-Dex, or Ixa-Dex<br>• IMiD-Dex<br><br>If > VGPR previously, consider treatment for rising dFLC only, before organ progression.	Optimal sequence unknown.<br><br>Options include PI, mAb, IMiD, venetoclax, bendamustine, HD melphalan + ASCT, belantamab.

a The consensus treatment guidelines for systemic AL amyloidosis recommend clinical trial participation for all patients at all stages, including newly diagnosed, ASCT, maintenance, and relapsed/refractory disease.
b Induction for Stage I-IIIa. Induction for Stage IIIb should be dose-modified Dara-CyBorD or single-agent daratumumab; alternatives include CyBorD or BMDex.
c Modification of treatment is recommended at a specified time point if the response is determined to be insufficient.

ASCO, American Society of Clinical Oncology; ASCT, autologous stem cell transplant; BMDex, bortezomib, melphalan, dexamethasone; CR, complete response; CyBorD, cyclophosphamide, bortezomib, dexamethasone; CYC, cyclophosphamide; Dara, daratumumab; Dex, dexamethasone; dFLC, difference in free light chains; EHA, European Haematology Association; FISH, fluorescent in situ hybridization; FLC, free light chain; hCR, hematologic complete response; HD, high dose; hVGPR, hematologic very good partial response; IFE, immunofixation electrophoresis; iFLC, involved free light chain; IMiD, immunomodulatory drug; ISA, International Society of Amyloidosis; Ixa, ixazomib; mAb, monoclonal antibody; Mel, melphalan; MM, multiple myeloma; mSMART, Mayo Stratification of Myeloma and Risk-Adapted Therapy; NCCN, National Comprehensive Cancer Network; OR, organ response; PI, proteasome inhibitor; uFLC, uninvolved free light chain; V-based, bortezomib-based; VGPR, very good partial response.

1. National Comprehensive Cancer Network. Systemic Light Chain Amyloidosis (v 2.2024) Available from: https://www.nccn.org/professionals/physician_gls/pdf/amyloidosis.pdf.
2. Dima D, Mazzoni S, Anwer F, Khouri J, Samaras C, Valent J, et al. Diagnostic and Treatment Strategies for AL Amyloidosis in an Era of Therapeutic Innovation. JCO Oncol Pract. 2023;19(5):265-75.
3. Muchtar E, Dispenzieri A, Gertz MA, Kumar SK, Buadi FK, Leung N, et al. Treatment of AL Amyloidosis: Mayo Stratification of Myeloma and Risk-Adapted Therapy (mSMART) Consensus Statement 2020 Update. Mayo Clinic Proceedings. 2021;96(6):1546-77.
4. Wechalekar AD, Cibeira MT, Gibbs SD, Jaccard A, Kumar S, Merlini G, et al. Guidelines for non-transplant chemotherapy for treatment of systemic AL amyloidosis: EHA-ISA working group. Amyloid. 2023;30(1):3-17.
5. Sanchorawala V, Boccadoro M, Gertz M, Hegenbart U, Kastritis E, Landau H, et al. Guidelines for high dose chemotherapy and stem cell transplantation for systemic AL amyloidosis: EHA-ISA working group guidelines. Amyloid. 2022;29(1):1-7.
