# Supplementary material for: Changing lanes: extending CAR T-cell therapy to high-risk plasma cell dyscrasias
Source: Front Immunol. 2025 Apr 8;16:1558275. doi: 10.3389/fimmu.2025.1558275 (PMC12011880; doi:10.3389/fimmu.2025.1558275)
Supplement: Supplementary file 1 [file DataSheet1.zip › Suppl Table 1A AL Clinical Trials.docx]

Supplemental Material Table 1A: Ongoing therapeutic clinical trials for AL amyloidosis (2014-2024).

| Drug Class | Study Regimen | Disease Setting | Phase | Target Enrollment | Clinicaltrials.gov Identifier | Study Start | Primary Completion | Status |
| --- | --- | --- | --- | --- | --- | --- | --- | --- |
| ASCT | ASCT, daratumumab, cyclophosphamide, bortezomib, dexamethasone | ND | 3 | 338 | NCT06022939 | 2024 | 2030 | Recruiting |
| Antibody Drug Conjugate | Belantamab Mafodotin | R/R | 1, 2 | 37 | NCT05145816 | 2024 | 2026 | Recruiting |
| Antibody Drug Conjugate | Belantamab Mafodotin | R/R | 2 | 35 | NCT04617925 | 2021 | 2025 | Active, not Recruiting |
| Antibody Drug Conjugate | STI-6129 | R/R | 1, 2 | 60 | NCT04316442 | 2021 | 2024 | Recruiting |
| Bispecific Ab | Linvoseltamab | R/R | 1, 2 | 220 | NCT06292780 | 2024 | 2028 | Recruiting |
| Bcl-2 Inhibitor | Venetoclax, daratumumab, dexamethasone | R/R | 1, 2 | 46 | NCT05486481 | 2024 | 2027 | Recruiting |
| Bcl-2 Inhibitor | Venetoclax, daratumumab, bortezomib, dexamethasone | ND, t(11;14) | IIT | 41 | NCT06192979 | 2024 | 2025 | Recruiting |
| Bcl-2 Inhibitor | Venetoclax, dexamethasone | ND, t(11;14) | 2 | 36 | NCT05996406 | 2024 | 2024 | Active, not Recruiting |
| Bcl-2 Inhibitor | Venetoclax, dexamethasone | R/R, t(11;14) | 1, 2 | 53 | NCT05451771 | 2022 | 2026 | Recruiting |
| Bcl-2 Inhibitor | Venetoclax, Ixazomib, dexamethasone | R/R | 1 | 24 | NCT04847453 | 2022 | 2026 | Recruiting |
| Bcl-2 Inhibitor | APG-2575, daratumumab, lenalidomide, dexamethasone | R/R | 1, 2 | 108 | NCT04942067 | 2021 | 2024 | Recruiting |
| BCMA T-cell Engager | Elranatamab monotherapy | R/R | 1, 2 | 49 | NCT06569147 | 2024 | 2027 | Not Yet Recruiting |
| BCMA T-cell Engager | ABBV-383 | R/R | 1, 2 | 76 | NCT06158854 | 2024 | 2025 | Recruiting |
| BCMA T-cell Engager | SAR445514 monotherapy | R/R | 1, 2 | 111^a^ | NCT05839626 | 2023 | 2026 | Recruiting |
| BCMA CAR-T | NXC-201 | R/R | 1 | 40 | NCT06097832 | 2024 | 2026 | Recruiting |
| BCMA CAR-T | FKC288 | R/R | 1 | 12 | NCT05978661 | 2023 | 2025 | Recruiting |
| IMID | Pomalidomide, bortezomib, dexamethasone | ND | 2 | 40 | NCT06342466 | 2024 | 2026 | Recruiting |
| Monoclonal Ab (anti-CD38) | Daratumumab, pomalidomide, dexamethasone | ND | IIT | 20 | NCT06455748 | 2024 | 2025 | Recruiting |
| Monoclonal Ab (anti-CD38) | Daratumumab, dexamethasone, pomalidomide, ASCT | ND | IIT | 100 | NCT06376214 | 2023 | 2025 | Recruiting |
| Monoclonal Ab (anti-CD38) | Daratumumab maintenance | 1L | 2 | 96 | NCT05898646 | 2023 | 2024 | Recruiting |
| Monoclonal Ab (anti-CD38) | Isatuximab, pomalidomide, dexamethasone | ≤ VGPR | 2 | 46 | NCT05066607 | 2022 | 2026 | Recruiting |
| Monoclonal Ab (anti-CD38) | Daratumumab, pomalidomide, dexamethasone | R/R | 2 | 21 | NCT04270175 | 2021 | 2025 | Recruiting |
| Monoclonal Ab (anti-CD38) | Isatuximab, bortezomib, cyclophosphamide, dexamethasone | ND, R/R HR | 1 | 11 | NCT04754945 | 2021 | 2024 | Active, not Recruiting |
| Monoclonal Ab (anti-CD38) | Daratumumab, pomalidomide | R/R | 2 | 40 | NCT04895917 | 2021 | 2024 | Active, not Recruiting |
| Monoclonal Ab (anti-CD38) | Daratumumab monotherapy | ND | 2 | 40 | NCT04131309 | 2019 | 2025 | Active, not Recruiting |
| Monoclonal Ab (anti-CD38) | Daratumumab, ixazomib, dexamethasone | ND | 1 | 21 | NCT03283917 | 2018 | 2025 | Active, not Recruiting |
| Monoclonal Ab (anti-CD38) | Isatuximab monotherapy | R/R | 2 | 43 | NCT03499808 | 2018 | 2023 | Active, not Recruiting |
| Monoclonal Ab (anti-CD38) | Daratumumab, bortezomib, cyclophosphamide, dexamethasone | ND | 3 | 416 | NCT03201965 | 2017 | 2020 | Active, not Recruiting |
| Monoclonal Ab (anti-fibril) | Birtamimab, cyclophosphamide, bortezomib, dexamethasone | ND, HR | 3 | 220 | NCT04973137 | 2021 | 2025 | Recruiting |
| Monoclonal Ab (anti-fibril) | CAEL-101 | ND | 3 | 124 | NCT04504825 | 2021 | 2024 | Active, not Recruiting |
| Monoclonal Ab (anti-fibril) | CAEL-101 | ND | 3 | 267 | NCT04512235 | 2020 | 2025 | Active, not Recruiting |
| Monoclonal Ab (anti-CS1) | Elotuzumab, lenalidomide, dexamethasone, cyclophosphamide | R/R | 2 | 53 | NCT03252600 | 2017 | 2023 | Active, not Recruiting |
| Proteosome Inhibitor | Ixazomib maintenance | ND | 2 | 17 | NCT03618537 | 2018 | 2026 | Active, not Recruiting |
| Trispecific Ab | JNJ-79635322 | R/R | 1 | 180 | NCT05652335 | 2022 | 2025 | Recruiting |

^a^ The trial enrollment included AL amyloid and MM patients.

1L, first line; Ab, antibody; ASCT, autologous stem cell transplant; BCMA, B-cell maturation antigen; HR, high risk; IIT, investigator-initiated trial; IMiD, immunomodulatory drug; ND, newly diagnosed; R/R, relapsed/refractory; VGPR, very good partial response.
