# Supplementary material for: Changing lanes: extending CAR T-cell therapy to high-risk plasma cell dyscrasias
Source: Front Immunol. 2025 Apr 8;16:1558275. doi: 10.3389/fimmu.2025.1558275 (PMC12011880; doi:10.3389/fimmu.2025.1558275)
Supplement: Supplementary file 1 [file DataSheet1.zip › Suppl Table 1B AL Clinical Trials.docx]

Supplemental Material Table 1B: Completed or inactive therapeutic clinical trials for AL amyloidosis (2014-2024).

| Drug Class | Study Regimen | Disease Setting | Phase | Target Enrollment | Clinicaltrials.gov Identifier | Study Start | Primary Completion | Status |
| --- | --- | --- | --- | --- | --- | --- | --- | --- |
| Antibody Drug Conjugate | STI-6129 | R/R | 1 | 0 | NCT05692908 | 2023 | 2024 | Withdrawn *^d^* |
| Bcl-2 Inhibitor | ZN-d5 | R/R | 1, 2 | 18 | NCT05199337 | 2021 | 2025 | Completed |
| Bcl-2 Inhibitor | Venetoclax, dexamethasone | R/R | 1 | 25 | NCT03000660 | 2017 | 2019 | Terminated *^a^* |
| BTK Inhibitor | Ibruitinib, bortezomib, dexamethasone | R/R | 2 | 0 | NCT03130348 | 2018 | 2022 | Withdrawn |
| Combination | Bortezomib, thalidomide, cyclophosphamide, dexamethasone | ND, R/R | 4 | 70 | NCT04612582 | 2020 | 2023 | Unknown |
| IMID | Pomalidomide, melphalan, dexamethasone | ND | 1 | 54 | NCT01807286 | 2014 | 2016 | Terminated |
| Monoclonal Ab (Anti-CD38) | Daratumumab monotherapy | TNE | IIT | 14 | NCT06571864 | 2022 | 2024 | Completed |
| Monoclonal Ab (Anti-CD38) | Isatuximab, bendamustine | ND TNE, R/R | 2 | 0 | NCT04943302 | 2022 | 2024 | Withdrawn *^c^* |
| Monoclonal Ab (Anti-CD38) | Daratumumab, bortezomib, dexamethasone | ND HR | 2 | 40 | NCT04474938 | 2021 | 2023 | Unknown |
| Monoclonal Ab (Anti-CD38) | Daratumumab | R/R | 1, 2 | 22 | NCT02841033 | 2017 | 2020 | Completed |
| Monoclonal Ab (Anti-CD38) | Daratumumab monotherapy | < VGPR | 2 | 40 | NCT02816476 | 2016 | 2019 | Completed |
| Monoclonal Ab (Anti-Fibril) | CAEL-101, bortezomib, cyclophosphamide, dexamethasone, daratumumab | ND, R/R | 2 | 25 | NCT04304144 | 2020 | 2023 | Completed |
| Monoclonal Ab (Anti-Fibril) | NEOD001 (Birtamimab) | R/R | 2 | 129 | NCT02632786 | 2016 | 2018 | Completed |
| Monoclonal Ab (Anti-Fibril) | NEOD001 (Birtamimab), bortezomib + SOC | ND | 3 | 236 | NCT02312206 | 2015 | 2018 | Terminated *^b^* |
| Monoclonal Ab (Anti-Fibril) | CAEL-101 | R/R, TNE | 1 | 31 | NCT02245867 | 2014 | 2017 | Completed |
| Peptidase-Enhanced Compound | Melphalan Flufenamide, dexamethasone | R/R | 1 | 46 | NCT04115956 | 2020 | 2022 | Terminated *^a^* |
| Proteasome Inhibitor | Ixazomib, cyclophosphamide, dexamethasone | ND | 1, 2 | 28 | NCT03236792 | 2017 | 2022 | Completed |
| Proteasome Inhibitor | Carfilzomib, thalidomide, dexamethasone | R/R | 1, 2 | 10 | NCT02545907 | 2017 | 2019 | Completed |
| Proteasome Inhibitor | HD Melphalan, ASCT, bortezomib | ND, R/R | 3 | 64 | NCT02489500 | 2015 | 2017 | Terminated |
| PI3K Inhibitor | Idelalisib | IgM ALA | 2 | 18 | NCT02590588 | 2016 | 2017 | Terminated *^e^* |
| SINE | Selinexor, dexamethasone | R/R | 1 | 0 | NCT04984330 | 2021 | 2023 | Withdrawn *^d^* |
| Tetracycline Antibiotics | Doxycycline, bortezomib, cyclophosphamide, dexamethasone | ND | IIT | 140 | NCT03401372 | 2018 | 2020 | Completed |

*a* Study terminated after a partial clinical hold by the US Food and Drug Administration.

*b* Study terminated due to futility.

*c* Study withdrawn due to hospital staffing issue.

d Study withdrawn due to funding.

*e* Study withdrawn due to poor accrual.

Ab, antibody; ALA, AL amyloidosis; BTK, Bruton’s tyrosine kinase; ND, newly diagnosed; HD, high dose; IgM, immunoglobulin M; HR, high risk; IIT, investigator-initiated trial; IMiD, immunomodulatory drug; PI3K, phosphoinositide 3-kinase; R/R, relapsed/refractory; SINE, selective-inhibitor-of-nuclear-export; SOC, standard of care; TNE, transplant not eligible; VGPR, very good partial response.
