# Supplementary material for: Changing lanes: extending CAR T-cell therapy to high-risk plasma cell dyscrasias
Source: Front Immunol. 2025 Apr 8;16:1558275. doi: 10.3389/fimmu.2025.1558275 (PMC12011880; doi:10.3389/fimmu.2025.1558275)
Supplement: Supplementary file 1 [file DataSheet1.zip › Suppl Table 2 PCL Clinical Trials.docx]

Supplemental Material Table 2: Therapeutic clinical trials enrolling pPCL (2014-2024).

| Study Intervention | Study Regimen | Disease Setting | Phase | Target Enrollment | Clinicaltrials.gov Identifier | Study Start | Primary Completion | | Status |
| --- | --- | --- | --- | --- | --- | --- | --- | --- | --- |
| *CAR-Based Trials* | | | | | | | | | |
| BCMA CAR-T ^a^ | **VRd + CART-ASCT-CART2** | **ND** | **IIT** | **20** | **NCT05870917** | **2023** | | **2025** | **Recruiting** |
| BCMA CAR-T ^a^ | **VRd + CAR-T** | **TNE** | **2** | **20** | **NCT05979363** | **2023** | | **2025** | **Recruiting** |
| BCMA CAR-T | CT0594CP CAR-T | R/R | IIT | 12 | NCT05219721 | 2023 | | 2026 | Recruiting |
| BCMA CAR-T | CT0594CP CAR-T | R/R | IIT | 6 | NCT05822037 | 2023 | | 2026 | Recruiting |
| GPRC5D CAR-T | CT071 CAR-T | R/R | IIT | 30 | NCT05838131 | 2023 | | 2024 | Recruiting |
| GPRC5D CAR-T | RD138 CAR-T | R/R | 1 | 12 | NCT05759793 | 2023 | | 2024 | Recruiting |
| BCMA CAR-NK | BCMA CAR-NK | R/R | IIT | 18 | NCT06045091 | 2023 | | 2025 | Recruiting |
| GPRC5D CAR-T | CT071 CAR-T | R/R | 1, 2 | 166 | NCT06333509 | 2024 | | 2027 | Not Yet Recruiting |
| GPRC5D CAR-T | GPRC5D CAR-T | R/R | 1 | 18 | NCT05219721 | 2022 | | 2024 | Unknown |
| *SCT-Based Trials* | | | | | | | | | |
| Induction + ASCT + Maintenance ^A^ | **Dara-VRd + ASCT #1, Dara-VRd consolidation #1, ASCT #2, Dara-VRD consolidation #2, lenalidomide maintenance** | **ND** | **2** | **29** | **NCT05054478** | **2021** | | **2024** | **Not Yet Recruiting** |
| Induction + ASCT + Maintenance | Dara-KRd-PACE + ASCT + Dara-KRd consolidation + Dara-Kd maintenance | ND | 2 | 54 | NCT06140966 | 2023 | | 2026 | Recruiting |
| Induction + SCT + Maintenance | ASCT or AlloSCT + PI-dex maintenance vs. PI + lenalidomide + dex consolidation + PI-dex maintenance | ND | IIT | 50 | NCT04008888 | 2018 | | 2020 | Unknown |
| JAK1-Inhibitor + PBSCT | Itacitinib + Non-myeloablative PBSCT + HD-cyclophosphamide | ³ PR | 1 | 32 | NCT05823571 | 2023 | | 2028 | Recruiting |
| ASCT | Mel + TMI + ASCT, lenalidomide maintenance | ³ PR | 1, 2 | -- | NCT03100877 | 2018 | | -- | Withdrawn |
| ALLO-SCT + Maintenance | BEAM AlloSCT + ixazomib maintenance | R/R | 1 | 11 | NCT02504359 | 2015 | | 2020 | Completed |
| Multiple Agents + ASCT | Panobinostat, gemcitabine, busulfan, melphalan + ASCT | R/R | 2 | 83 | NCT02506959 | 2015 | | 2024 | Completed |
| *Combination Regimen Trials* | | | | | | | | | |
| Multiple Agents ^A^ | **Dara-Vd +pegylated liposomal doxorubicin + lenalidomide** | **ND, R/R** | **1** | **--** | **NCT03591744** | **2018** | | **--** | **Withdrawn** |
| Multiple Agents | Dara-VRd + low-dose cyclophosphamide | ND | 2 | 95 | NCT03188172 | 2017 | | 2025 | Active, not Recruiting |
| Multiple Agents | Isatuximab + dexamethasone, lenalidomide | ND | 2 | 40 | NCT06517017 | 2024 | | 2026 | Not Yet Recruiting |
| Multiple Agents | Ixazomib, pomolidomide, dexamethasone | R/R | 2 | 17 | NCT02547662 | 2015 | | 2020 | Completed |

^a^ Most studies are currently enrolling patients with PCL and various other plasma cell disorders. However, those studies highlighted in bold are exclusively enrolling patients with PCL.

AlloSCT, allogeneic stem cell transplant; ASCT, autologous stem cell transplant; BCMA, B-cell maturation antigen; BEAM, carmustine, etoposide, cytarabine, melphalan; CAR-NK, chimeric antigen receptor NK cell; CAR-T, CART, chimeric antigen receptor T-cell therapy; Dara, daratumumab; dex, dexamethasone; HD, high dose; IIT, investigator-initiated trial; JAK1, Janus kinase 1; Kd, carfilzomib, dexamethasone; KRd, carfilzomib, lenalidomide, dexamethasone; Mel, melphalan; ND, newly diagnosed; PACE, cisplatin, doxorubicin, cyclophosphamide, etoposide; PBSCT, peripheral blood stem cell transplant; PI, proteasome inhibitor; pPCL, primary plasma cell leukemia; PR, partial response; R/R, relapsed/refractory; SCT, stem cell transplant; TMI, total marrow irradiation; TNE, transplant not eligible; Vd, bortezomib, dexamethasone. VRd, bortezomib, lenalidomide, dexamethasone.
