# Supplementary material for: Changing lanes: extending CAR T-cell therapy to high-risk plasma cell dyscrasias
Source: Front Immunol. 2025 Apr 8;16:1558275. doi: 10.3389/fimmu.2025.1558275 (PMC12011880; doi:10.3389/fimmu.2025.1558275)
Supplement: Supplementary file 1 [file DataSheet1.zip › Suppl Table 3A Safety Summary by Study AL.docx]

Supplemental Material Table 3A: Safety event summary for AL amyloidosis by publication.

| Reference | N | Adverse Event (N) | Any Grade  N (% N by study) | G1-2 | G3-4 | G5 |
| --- | --- | --- | --- | --- | --- | --- |
| (1-4) Lebel et al | **16** |  |  |  |  |  |
|  |  | CRS | 14 (88%) | 11 | 3 |  |
|  |  | ICANS | 0 (0%) | 0 | 0 |  |
|  |  | Hematologic  Neutropenia  Lymphopenia  Anemia  Thrombocytopenia | 12 (75%)  16 (100%)  12 (75%)  9 (56%) | 2  0  7  9 | 10  16  5  0 |  |
|  |  | CHF Exacerbation | 3 (19%) | 0 | 3 |  |
|  |  | Acute kidney injury | 4 (25%) | 4 | 0 |  |
|  |  | Hepatic dysfunction | 6 (38%) | 2 | 4 |  |
|  |  | Infections  Febrile neutropenia  Early infections (≤ day +28)  Late infections (> day +28)  Pneumonia  COVID-19  Infectious diarrhea  Osteomyelitis | 5 (31%)  9 (56%)  7 (44%)  6 (38%)  2 (13%)  2 (13%)  1 (6%) | 0  3  Unk | 5  6  Unk |  |
|  |  | Cardiac disease | 5 (31%) |  |  | 5 |
|  |  | Thrombosis (PE, DVT) | 2 (13%) |  |  |  |
|  |  | Myelodysplastic syndrome | 1 (6%) |  |  |  |
|  |  | Worsening depression | 1 (6%) |  |  |  |
|  |  | Pruritis | 1 (6%) | 1 | 0 |  |
|  |  | Drug toxicity | 1 (6%) |  |  |  |
| (5) Goel et al. | **8** |  |  |  |  |  |
|  |  | CRS | 6 (75%) | 6 | 0 |  |
|  |  | ICANS | 1 (12.5%) | 1 | 0 |  |
|  |  | Hematologic *^a^*  Cytopenia (d+30) | Unk  7 (87%) |  |  |  |
|  |  | Neutropenia | 5 (62.5%) |  |  |  |
|  |  | Anemia | 3 (37.5%) |  |  |  |
|  |  | Thrombocytopenia | 2 (25%) |  |  |  |
|  |  | Infections |  |  |  |  |
|  |  | Respiratory viral infection | 3 (37.5%) | 2 | 1 |  |
| (6) Das et al. | **2** |  |  |  |  |  |
|  |  | CRS | 1 (50%) |  | 1 |  |
|  |  | ICANS | 0 (0%) |  |  |  |
|  |  | Hematologic | 2 (100%) |  |  |  |
|  |  | Neutropenia | 2 (100%) |  | 2 |  |
|  |  | Anemia | 2 (100%) | 2 |  |  |
|  |  | Thrombocytopenia | 1 (50%) |  | 1 |  |
| (7) Oliver-Caldes et al. | **1** |  |  |  |  |  |
|  |  | CRS | 1 (100%) | 1 |  |  |
|  |  | ICANS | 0 (0%) |  |  |  |
|  |  | Hematologic | 1 (100%) |  |  |  |
|  |  | Neutropenia | 1 (100%) |  | 1 |  |
|  |  | Thrombocytopenia | 1 (100%) |  | 1 |  |
|  |  | Infections | 1 (100%) |  |  |  |
|  |  | SARS-CoV-2 pneumonia | 1 (100%) |  | 1 |  |
|  |  | BK virus hemorrhagic cystitis | 1 (100%) |  | 1 |  |

Abbreviations: AL, AL amyloidosis; CHF, congestive heart failure; CRS, cytokine release syndrome; d, day; DVT, deep vein thrombosis; G, grade; GI, gastrointestinal; ICANS, immune effector cell associated neurotoxicity syndrome; N, number of patients; PCL, plasma cell leukemia; pPCL, primary plasma cell leukemia, SARS-CoV2, severe acute respiratory syndrome coronavirus 2; sPCL, secondary plasma cell leukemia; Unk, unknown.

^a^ Authors reported adverse events cumulatively as “any grade”, without providing data on quantities of categorical grades. Cumulative total events reported here with this limitation.

### REFERENCES

1. Lebel E, Asherie N, Kfir-Erenfeld S, Grisariu S, Avni B, Elias S, et al. Efficacy and Safety of Anti–B-Cell Maturation Antigen Chimeric Antigen Receptor T-Cell for the Treatment of Relapsed and Refractory AL Amyloidosis. Journal of Clinical Oncology. 2024;0(0):JCO-24-02252.

2. Lebel E, Kfir-Erenfeld S, Asherie N, Grisariu S, Avni B, Elias S, et al. Feasibility of a Novel Academic Anti-BCMA Chimeric Antigen Receptor T-Cell (CART) (HBI0101) for the Treatment of Relapsed and Refractory AL Amyloidosis. Blood. 2023;142:538-9.

3. Kfir-Erenfeld S, Asherie N, Grisariu S, Avni B, Zimran E, Assayag M, et al. Feasibility of a Novel Academic BCMA-CART (HBI0101) for the Treatment of Relapsed and Refractory AL Amyloidosis. Clin Cancer Res. 2022;28(23):5156-66.

4. Haran A, Vaxman I, Gatt ME, Lebel E. Immune Therapies in AL Amyloidosis-A Glimpse to the Future. Cancers (Basel). 2024;16(8).

5. Goel U, Dima D, Davis J, Ahmed N, Shaikh H, Lochner J, et al. Safety and efficacy of B cell maturation antigen-directed CAR T-cell therapy in patients with relapsed/refractory multiple myeloma and concurrent light chain amyloidosis. Eur J Haematol. 2024:1-7.

6. Das S, Ailawadhi S, Sher T, Roy V, Fernandez A, Parrondo RD. Anti-B Cell Maturation Antigen Chimeric Antigen Receptor T Cell Therapy for the Treatment of AL Amyloidosis and Concurrent Relapsed/Refractory Multiple Myeloma: Preliminary Efficacy and Safety. Curr Oncol. 2023;30(11):9627-33.

7. Oliver-Caldes A, Jimenez R, Espanol-Rego M, Cibeira MT, Ortiz-Maldonado V, Quintana LF, et al. First report of CART treatment in AL amyloidosis and relapsed/refractory multiple myeloma. J Immunother Cancer. 2021;9(12):e003783.
